# A RUBY-Assisted Hairy Root Transformation Platform for Metabolomic Profiling and Preliminary Functional Analysis of *HpRHL76* in *Herpetospermum pedunculosum*

**DOI:** 10.3390/plants15162514

**Published:** 2026-08-20

**Authors:** Shiyu Yao, Yujie Yang, Jinyue Wang, Xinghui Wu, Mingrong Liu, Min Sun, Ziwei Zhu, Bingliang Liu, Qiuxia Lu, Yixi Yang, Rui Li, Qi Zhao, Yang Tao

**Affiliations:** 1 Engineering Research Center of Sichuan-Xizang Traditional Medicinal Plant, College of Food and Biological Engineering, Chengdu University, Chengdu 610106, China; 19181755089@163.com (S.Y.); wangjin_yue@163.com (J.W.); wuxinghui3949@163.com (X.W.); 202310711225@cdu.edu.cn (M.L.); summin1028@163.com (M.S.); zhuziwei@cdu.edu.cn (Z.Z.); liubingliang@cdu.edu.cn (B.L.); luqiuxia@cdu.edu.cn (Q.L.); yangyixi1011@cdu.edu.cn (Y.Y.); lirui@cdu.edu.cn (R.L.); 2 Sichuan-Xizang Medicinal Resource Breeding and Standardization Team, Institute for Advanced Study, Chengdu University, Chengdu 610106, China; 3 Institute of Urban Agriculture, Chinese Academy of Agricultural Sciences, National Agricultural Science & Technology Center, Chengdu 610213, China; yangyujie@caas.cn; 4 Natural Products Chem-Bio Innovation Center, Chengdu University, Chengdu 610106, China

**Keywords:** *Herpetospermum pedunculosum*, RUBY reporter, *Rhizobium rhizogenes*, widely targeted metabolomics, metabolic engineering, secondary metabolism, root hair development

## Abstract

*Herpetospermum pedunculosum* is an endangered cucurbitaceous medicinal plant whose functional genomic and metabolic studies are constrained by the lack of efficient aseptic seedling and genetic transformation systems. Here, we established a rapid aseptic seedling production method and a RUBY-assisted hairy root transformation platform integrated with metabolomic evaluation. The cotton-supported liquid culture method provided a favorable balance between germination performance and experimental operability. Microsyringe-mediated stem-base inoculation with *Rhizobium rhizogenes* K599 carrying 35S::RUBY enabled rapid visual screening of RUBY-positive hairy roots, with a maximum transformation efficiency of 93.33%. Widely targeted metabolomics identified 242 metabolites and revealed major differences in amino acid/nitrogen and sugar metabolism, lipid-related metabolism, specialized metabolite biosynthesis, hormone-related pathways, and ABC transporters among wild-type, vector-free K599-induced, and RUBY-expressing roots. In addition, 80 HpRHL-like bHLH genes were identified. As a proof of concept, RT-qPCR confirmed *HpRHL76* overexpression, which was associated with an increase in mean root hair length from approximately 0.4 to 1.1 mm. Together, this integrated framework provides a practical basis for preliminary candidate gene assessment, functional genomic studies, and future metabolic engineering of medicinally relevant compounds in *H. pedunculosum*, and may serve as a methodological reference for other difficult-to-transform medicinal plants.

## 1. Introduction

*Herpetospermum pedunculosum* (Ser.) C.B. Clarke is a high-altitude medicinal plant of the Cucurbitaceae family and is widely used in traditional Tibetan medicine. Its dried ripe seeds are the principal medicinal material and contain diverse specialized metabolites, among which lignans represent one of the most characteristic and pharmacologically important classes [1,2]. Several lignans, including herpetin, herpetrione, herpetotriol, and related compounds, have been associated with hepatoprotective, anti-cholestatic, anti-inflammatory, and antioxidant activities [3,4]. Terpenoids, including cucurbitacin derivatives, as well as flavonoids and other phenolic compounds, have also been reported in this species and may contribute to its pharmacological properties [3,4]. These metabolites make *H. pedunculosum* an important medicinal resource and provide potential targets for biosynthetic and metabolic engineering studies. However, wild populations are sparsely distributed in alpine habitats and are threatened by habitat degradation and excessive harvesting [5,6]. The species is also dioecious, has a relatively low natural proportion of female plants [7,8], and low conventional propagation efficiency [9]. Moreover, an efficient and stable whole-plant genetic transformation system has not yet been established, limiting rapid functional validation of genes involved in the biosynthesis and regulation of its medicinally relevant metabolites.

For plant species in which stable transformation remains difficult, hairy root transformation provides a useful alternative for gene functional analysis, specialized metabolite studies, and metabolic engineering [10,11]. Hairy roots generated through *Rhizobium rhizogenes* (formerly *Agrobacterium rhizogenes*)-mediated transformation exhibit rapid, hormone-independent growth and can maintain stable production of specialized metabolites [12]. Compared with whole-plant regeneration, hairy root induction generally requires a shorter experimental cycle and avoids complex regeneration procedures [13]. Hairy root systems have therefore been successfully applied to a wide range of recalcitrant and medicinal plants for both gene validation and manipulation of metabolite accumulation [14,15]. For example, overexpression of *SmGGPPS* and *SmDXSII* in *Salvia miltiorrhiza* hairy roots increased total tanshinone accumulation to 12.93 mg/g dry weight [16]. Nevertheless, hairy roots are not metabolically neutral materials. The *rol* genes introduced through Ri T-DNA can alter endogenous hormone homeostasis, development, and metabolic processes, and the metabolic profiles of in vitro hairy roots may not fully represent those of intact roots or whole plants [17,18]. Therefore, transformation-associated metabolic changes should be considered when hairy roots are used for functional or metabolic studies.

Efficient identification of transformed roots is another important requirement for establishing a practical hairy root system [19]. Conventional reporter systems such as GFP, YFP, DsRed, and GUS are widely used, but fluorescent reporters require specialized imaging equipment, whereas GUS detection involves destructive histochemical staining and does not permit continuous observation of the same living tissue. Antibiotic- or herbicide-based selection may further prolong the screening process [20,21]. The betalain biosynthesis-based RUBY reporter provides a convenient alternative because transformed tissues accumulate visible red pigments that can be recognized directly without fluorescence instrumentation or destructive staining [22,23]. RUBY has been successfully applied to hairy root transformation in several plant species, including soybean and mung bean [24,25]. However, because RUBY introduces an active betalain biosynthetic pathway that utilizes endogenous tyrosine, its expression may itself contribute to the metabolic background of transformed tissues. Together with the metabolic effects of *R. rhizogenes*-mediated transformation, this highlights the need to distinguish hairy root- and RUBY-associated metabolic variation when developing such a platform for metabolic studies. Relatively few studies have integrated species-adapted aseptic seedling establishment, visual screening of transformed roots, and comparative assessment of transformation-associated metabolic backgrounds within a single workflow. Such an integrated system has not previously been established for *H. pedunculosum*.

To further evaluate whether a newly established hairy root system could support preliminary functional analysis, root hair development provides a readily observable proof-of-concept phenotype. The basic helix-loop-helix (bHLH) family is one of the largest transcription factor families in plants and participates broadly in development, hormone signaling, stress responses, and specialized metabolism [26]. RHL-related bHLH factors are closely associated with root hair development, and functional disruption of RHL homologs in *Arabidopsis*, melon, and *Lotus japonicus* results in clear root hair phenotypes [27,28,29,30]. These characteristics make RHL-related genes suitable candidates for evaluating a hairy root-based functional validation system.

Based on these considerations, we hypothesized that integrating RUBY-based visual screening with evaluation of the transformation-associated metabolic background could provide a practical and more interpretable hairy root platform for *H. pedunculosum*. Accordingly, we established and optimized an aseptic seedling production and RUBY-assisted hairy root transformation workflow and compared the metabolic profiles of wild-type roots, vector-free K599-induced hairy roots, and RUBY-expressing hairy roots. We also performed a genome-wide identification of HpRHL-like bHLH genes. Among the identified candidates, *HpRHL76* was selected as a proof-of-concept target based on its phylogenetic proximity to functionally characterized RHL proteins and its relatively high expression in roots and was subsequently used to evaluate the applicability of the platform for root development-related gene analysis. This integrated framework provides a practical experimental basis for future functional validation of candidate genes and investigation of specialized metabolite biosynthesis in *H. pedunculosum*.

## 2. Results

### 2.1. Optimization of Aseptic Seedling Establishment in H. pedunculosum

To obtain aseptic explants suitable for subsequent hairy root induction, we compared three methods for seed germination and aseptic seedling establishment in *H. pedunculosum*: the solid medium method (Sm), vermiculite medium method (Vm), and cotton-supported liquid culture method (Clm). The conventional Sm method was associated with low germination efficiency and a long germination period, and it usually required removal of the seed coat to promote germination, which increased the time required for pretreatment and the risk of contamination. To improve the efficiency of aseptic seedling production, we established Vm and Clm as two alternative culture methods and systematically compared their germination performance and practical applicability.

**Figure 1:**
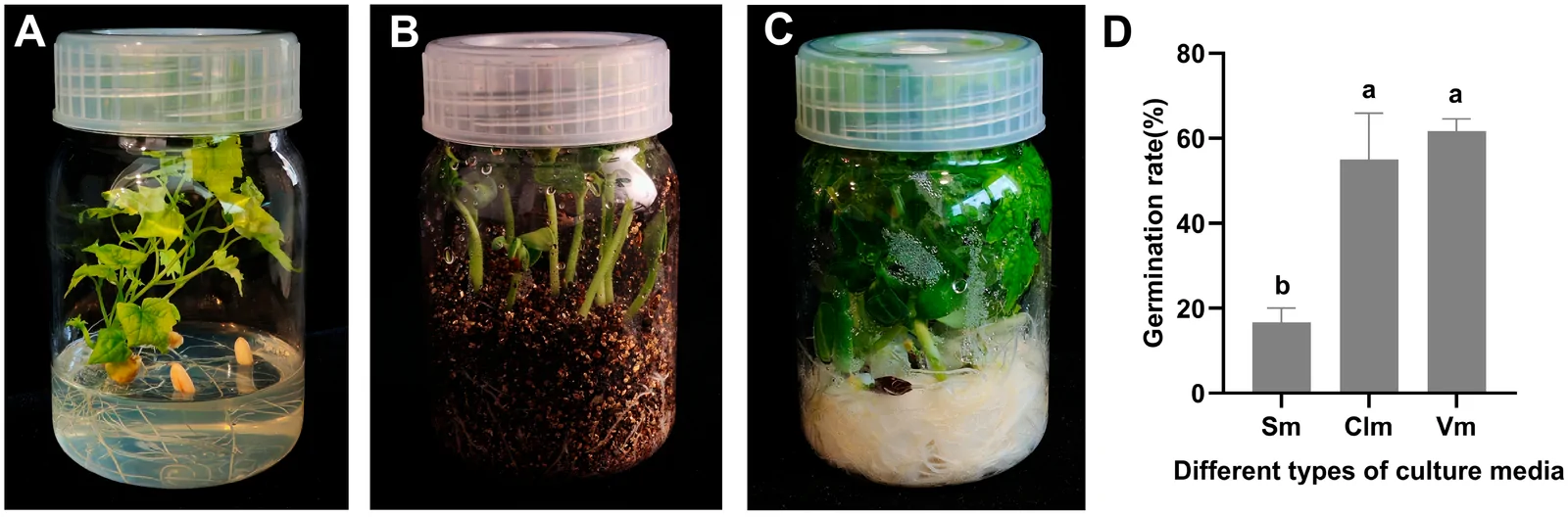
Aseptic seedling establishment of *H. pedunculosum* under different culture substrates. (**A**) Solid medium method (Sm). (**B**) Vermiculite medium method (Vm). (**C**) Cotton-supported liquid culture method (Clm). (**D**) Effects of different culture methods on the seed germination rate of *H. pedunculosum*. Different lowercase letters indicate significant differences at *p* < 0.05.

The three culture methods showed clear differences in germination time, germination rate, and ease of operation and contamination control (Figure 1). Seeds cultured using Sm germinated most slowly, usually after 15–20 d, with a germination rate of only 17.78 ± 3.33%. This method was also associated with a relatively high contamination rate and apparent germination, making it unsuitable for subsequent experiments. In contrast, Clm significantly improved seed germination efficiency, shortening the germination period to 10–15 d and increasing the germination rate to 54.89 ± 10.23%. In addition, contamination was easier to observe in Clm, allowing early identification and removal of contaminated materials. Vm showed the best numerical germination performance, with the shortest germination period of 8–13 d and the highest germination rate of 61.67 ± 2.89%. However, because vermiculite is an opaque substrate, latent contamination could not be readily detected at an early stage, which may increase the risk of cross-contamination during subsequent culture.

Notably, there was no significant difference in germination rate between Clm and Vm (Figure 1D), whereas Clm provided better contamination visibility and ease of operation and contamination control, facilitating the selection of truly aseptic seedlings for subsequent hairy root induction. Therefore, considering germination efficiency, contamination control, operational convenience, and compatibility with downstream genetic transformation, Clm was selected as a suitable method for aseptic seedling establishment and hairy root induction in *H. pedunculosum*.

### 2.2. Optimization of RUBY-Assisted Hairy Root Induction in H. pedunculosum

To our knowledge, no hairy root induction system has yet been reported for *H. pedunculosum*. To establish an efficient hairy root transformation system for this species, we used *R. rhizogenes* strain K599 carrying the 35S::RUBY reporter to induce hairy roots from aseptic seedlings and systematically optimized key parameters, including inoculation method, bacterial suspension volume, cefotaxime concentration, and bacterial density.

**Figure 2:**
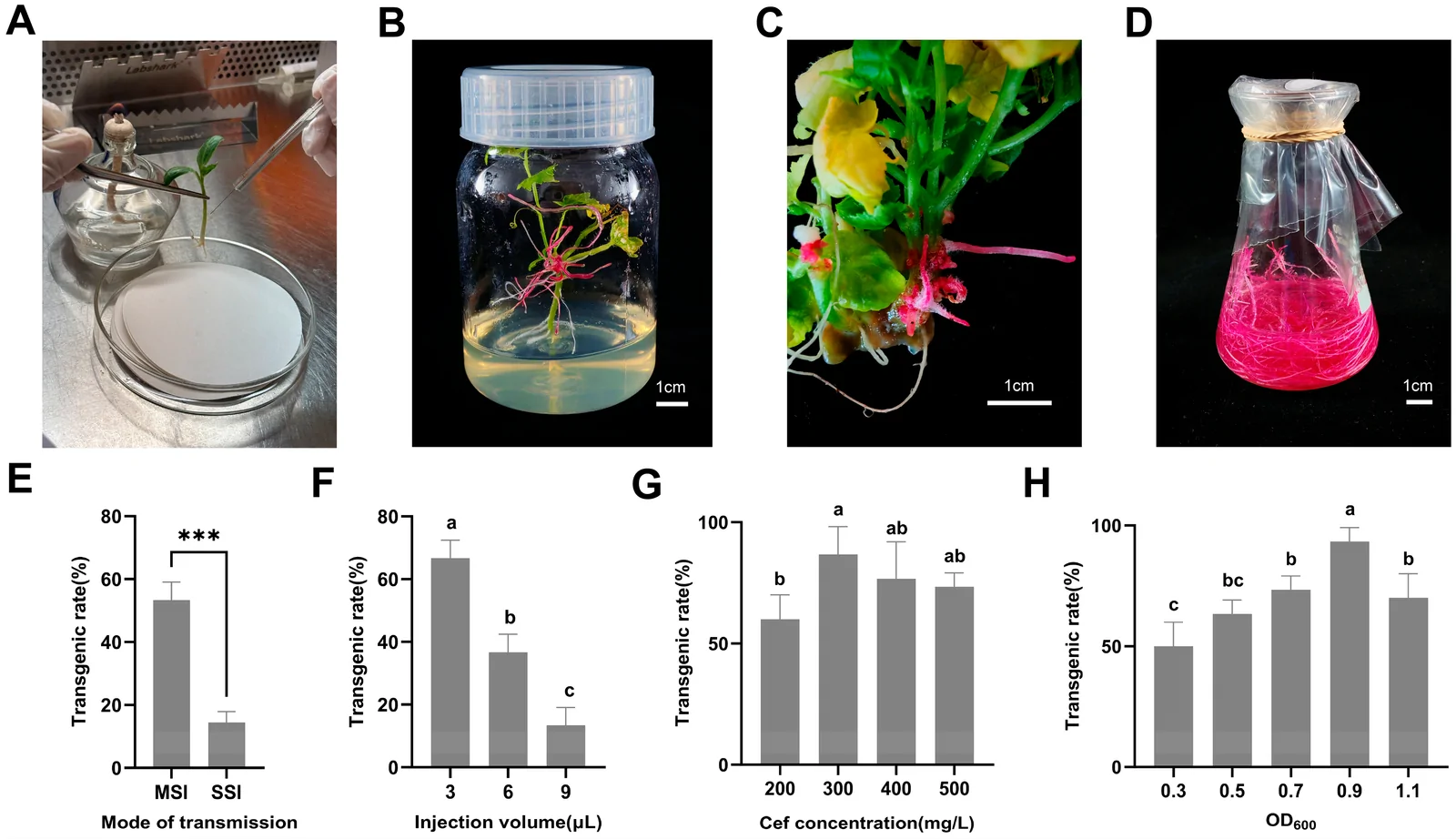
Establishment of a RUBY-labeled hairy root transformation system in *H. pedunculosum*. (**A**–**D**) Hairy root induction in *H. pedunculosum* using microsyringe-mediated stem-base inoculation. (**A**) Injection treatment of aseptic seedlings. (**B**,**C**) Red RUBY-positive hairy roots induced 30 d after inoculation. (**D**) Hairy roots transferred to liquid medium for continued culture on a shaker. Quantitative analysis of hairy root transformation efficiency under different conditions: (**E**) comparison of inoculation methods; (**F**) different injection volumes; (**G**) different cefotaxime sodium concentrations; and (**H**) different bacterial densities. Each treatment contained 10 individual seedlings per biological replicate, with three independent biological replicates performed. Error bars represent the standard error of the mean (SEM). Asterisks (***) in (**E**) indicate a significant difference at *p* < 0.001 (Student’s *t*-test). Different lowercase letters in (**F**–**H**) indicate significant differences at *p* < 0.05 (one-way ANOVA with Tukey’s multiple comparison test).

First, we compared two inoculation methods: microsyringe-mediated stem-base inoculation (MSI) (Figure 2A) and wounded stem-segment immersion (SSI). In the MSI method, the bacterial suspension was directly delivered into the stem base of aseptic seedlings using a sterile microsyringe, and visible red RUBY-positive hairy roots could be observed after 3–4 weeks of induction (Figure 2B). The results showed that MSI achieved a hairy root transformation efficiency of 52.78 ± 5.09%, which was significantly higher than that of SSI (13.89 ± 2.78%) (*p* < 0.001, Figure 2E). Under SSI treatment, only a few hairy roots were induced at the cut sites of hypocotyl or stem segments, and the explants were more prone to contamination or death. By contrast, seedlings treated with MSI showed better overall survival and a higher hairy root transformation efficiency. Therefore, MSI was used in all subsequent optimization experiments.

Further optimization showed that the volume of bacterial suspension had a significant effect on hairy root transformation efficiency. When the injected volume was 3 μL, the transformation efficiency reached the highest value of 66.67% (Figure 2F). As the injection volume increased further, the transformation efficiency declined markedly, suggesting that excessive bacterial suspension may inhibit explant growth and hairy root formation. Therefore, 3 μL was selected as the appropriate injection volume for subsequent hairy root induction experiments.

To suppress excessive growth of K599 while minimizing its adverse effects on explants, we further evaluated the effect of different concentrations of cefotaxime sodium (Cef) on hairy root induction. The highest transformation efficiency, 87.67%, was obtained on 1/2 MS medium supplemented with 300 mg/L Cef (Figure 2G). At lower Cef concentrations, bacterial elimination was insufficient and interfered with subsequent culture, whereas higher Cef concentrations reduced hairy root transformation efficiency, indicating that excessive antibiotic levels may be unfavorable for hairy root formation.

Bacterial density also had a marked effect on hairy root transformation efficiency in *H. pedunculosum*. As the bacterial density increased from OD600 = 0.3 to OD600 = 0.9, the transformation efficiency increased progressively and reached a maximum of 93.33% at OD600 = 0.9, which was significantly higher than that of the other treatments (*p* < 0.05, Figure 2H). When the bacterial density was further increased to OD600 = 1.1, the transformation efficiency decreased to 70.00%, and explants were more likely to exhibit browning or growth inhibition. Taken together, the optimal conditions for RUBY-assisted hairy root induction in *H. pedunculosum* were MSI as the inoculation method, an injection volume of 3 μL, 300 mg/L Cef, and a bacterial density of OD600 = 0.9. Under these conditions, RUBY-positive hairy roots could be efficiently induced, providing a useful basis for subsequent transgenic hairy root screening and gene functional analysis.

### 2.3. Metabolomic Profiling Reveals Broad Metabolic Changes Associated with the Hairy Root State

**Figure 3:**
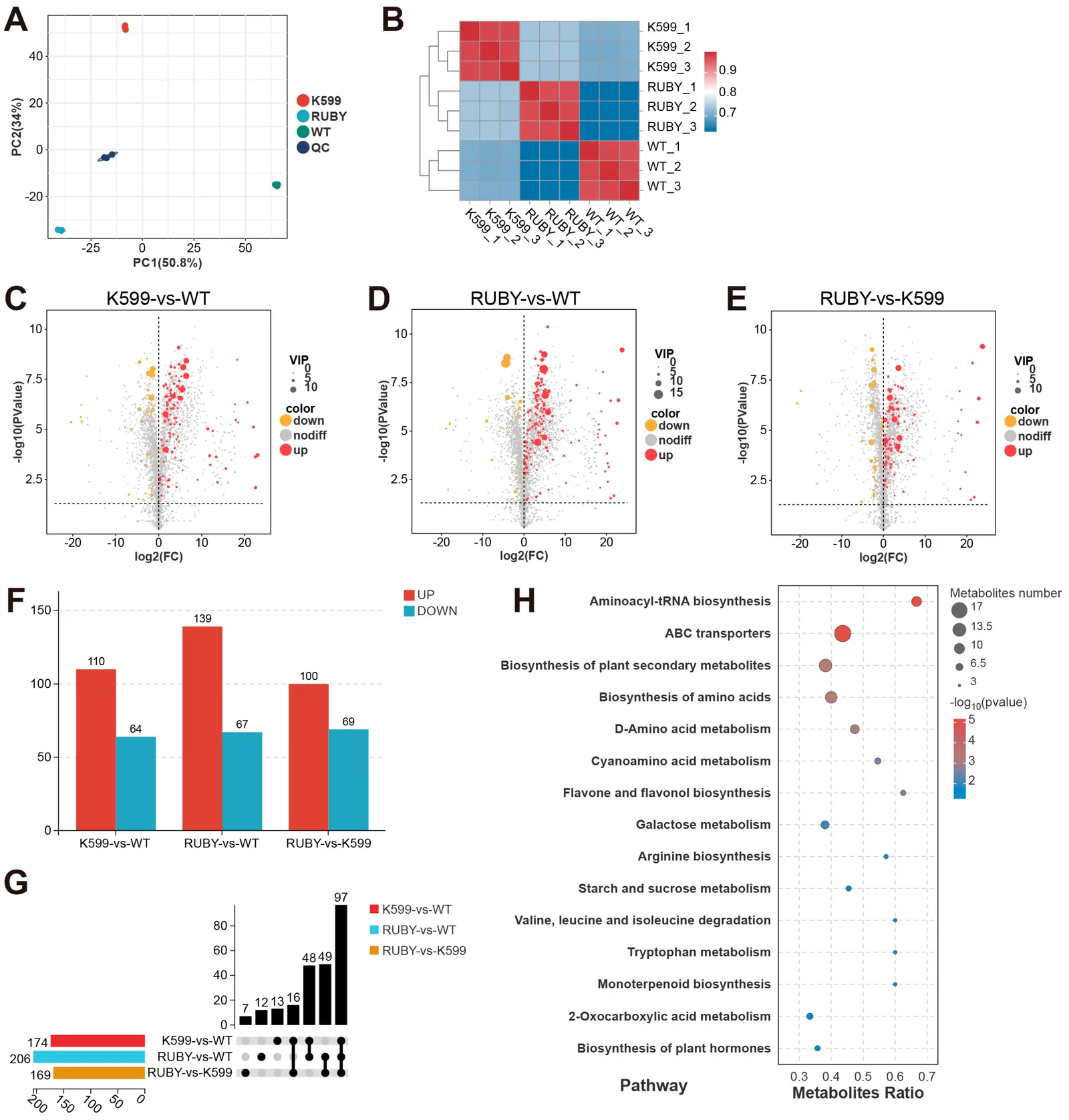
Widely targeted metabolomic profiling reveals broad metabolic changes associated with the hairy root state in *H. pedunculosum*. (**A**) Principal component analysis (PCA) of metabolomic profiles from wild-type roots (WT), vector-free K599-induced hairy roots (K599), RUBY-expressing hairy roots (RUBY), and quality control samples (QC). (**B**) Pearson correlation heatmap of metabolomic profiles among WT, K599, and RUBY samples. (**C**–**E**) Volcano plots showing differential metabolites in K599 vs. WT (**C**), RUBY vs. WT (**D**), and RUBY vs. K599 (**E**). (**F**) Number of up- and down-regulated differential metabolites identified in the three pairwise comparisons. (**G**) UpSet plot showing shared and comparison-specific differential metabolites among K599 vs. WT, RUBY vs. WT, and RUBY vs. K599. (**H**) KEGG pathway enrichment analysis of HRs vs. WT differential metabolites. Bubble size represents the number of enriched metabolites, and color indicates the enrichment significance.

Widely targeted metabolomic profiling was performed on WT (wild-type roots), K599 (hairy roots induced by *R. rhizogenes* K599 without an introduced binary vector), and RUBY (hairy roots induced by *R. rhizogenes* K599 carrying the 35S::RUBY construct) samples. The QC samples clustered tightly, indicating good analytical stability and reproducibility. Principal component analysis (PCA) showed clear separation among the three sample groups (Figure 3A). Both K599 and RUBY samples were clearly separated from WT roots, indicating substantial metabolic differences associated with the hairy root state. RUBY samples were also distinguishable from K599 samples, indicating additional metabolic differences associated with the introduction of the RUBY construct and/or RUBY expression. The sample correlation heatmap further supported good reproducibility within each group (Figure 3B).

A total of 242 metabolites were annotated across the three sample groups. Pairwise comparisons identified 174 differential metabolites in K599 vs. WT and 206 differential metabolites in RUBY vs. WT (Figure 3C–F), indicating extensive metabolic changes associated with hairy root formation and the transformation background. Consistent with a previous study in pumpkin comparing WT roots, empty-vector hairy roots, and transgenic hairy roots, hairy root formation itself can induce substantial transcriptomic and metabolomic changes [31]. In addition, 169 differential metabolites were detected in RUBY vs. K599 (Figure 3F), suggesting additional metabolic variation associated with the introduction of the RUBY construct and/or RUBY expression, with up-regulated metabolites predominating in all three comparisons. UpSet analysis revealed 97 differential metabolites shared across all three pairwise comparisons, together with additional comparison-specific metabolites, indicating both broadly responsive metabolites and distinct metabolic differences among the three comparisons (Figure 3G).

Because all three pairwise comparisons were analyzed separately, pooling K599 and RUBY samples as the HRs group was used only as a complementary analysis to characterize the overall metabolic background associated with the hairy root state, rather than to evaluate differences between the two hairy root types. K599 and RUBY samples were therefore pooled as the HRs group and compared with WT roots to obtain an overall view of hairy-root-associated metabolic changes. Four major metabolite classes, including sugars, organic acids, amino acids, and lipids, were further examined (Table S2). A total of 80 differential metabolites were identified within these four classes. Amino acids represented the largest category, with 37 differential metabolites, followed by sugars with 25, organic acids with 13, and lipids with 5. Among the four major metabolite classes examined, amino acid-related metabolites showed the most pronounced changes, with most displaying increased accumulation in HRs. Representative examples included L-arginine, L-proline, and L-glutamine. In contrast, several monosaccharides decreased, whereas several disaccharides and oligosaccharides accumulated. Organic acids showed bidirectional changes, while several lipid-related metabolites were reduced (Table S2).

KEGG enrichment analysis showed that the differential metabolites were mainly associated with amino acid metabolism, carbohydrate metabolism, flavone and flavonol biosynthesis, monoterpenoid biosynthesis, plant hormone biosynthesis, and ABC transporters (Figure 3H). Taken together, the overall metabolic profile of HRs relative to WT roots was characterized by broad changes in amino acid/nitrogen-related metabolism, redistribution of sugar metabolites, decreases in several membrane lipid-related metabolites, and changes in pathways associated with flavonoids, monoterpenoids, plant hormones, and metabolite transport.

### 2.4. Selected Specialized and Stress-Related Metabolites Differ Among WT, K599, and RUBY Roots

**Figure 4:**
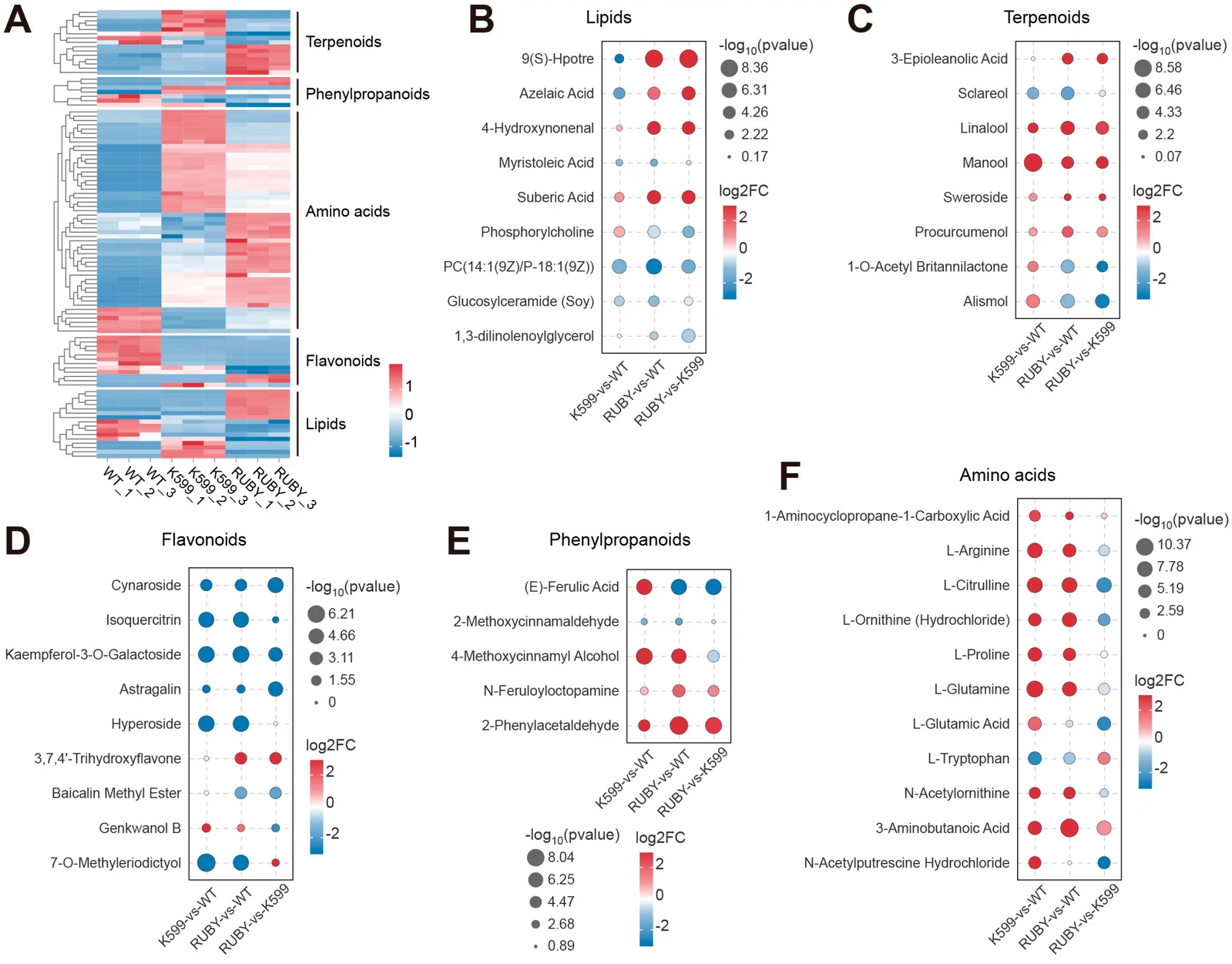
Comparative analysis of selected specialized and stress-related metabolites among WT, K599, and RUBY roots of *H. pedunculosum*. (**A**) Heatmap showing the relative accumulation patterns of selected differential metabolites belonging to five major metabolite categories, including lipids, terpenoids, flavonoids, phenylpropanoids, and amino acids, in WT, K599, and RUBY samples. (**B**–**F**) Bubble plots showing the log_2_ fold changes and statistical significance of representative metabolites in K599 vs. WT, RUBY vs. WT, and RUBY vs. K599 comparisons. The selected metabolites include lipids (**B**), terpenoids (**C**), flavonoids (**D**), phenylpropanoids (**E**), and amino acids (**F**). In the bubble plots, colors indicate log_2_ fold changes, and bubble sizes indicate statistical significance. WT, wild-type roots; K599, vector-free K599-induced hairy roots; RUBY, RUBY-expressing hairy roots.

We next focused on differential metabolite categories associated with specialized metabolism, transformation-related stress, and RUBY expression. Five major metabolite classes, including terpenoids, phenylpropanoids, amino acids, flavonoids, and lipids, were extracted from the differential metabolites for further analysis (Table S3). Heatmap analysis showed that WT, K599, and RUBY samples exhibited distinct metabolic states (Figure 4A). Representative metabolites from these five classes were further selected to compare their log2 fold changes and statistical significance in K599 vs. WT, RUBY vs. WT, and RUBY vs. K599, and the results were visualized using bubble plots (Figure 4B–F). Different metabolite classes showed distinct response patterns across the three comparisons.

Among lipid-related metabolites, suberic acid, azelaic acid, 9(S)-HpOTrE, and 4-hydroxynonenal showed more pronounced changes in the RUBY group (Figure 4B), whereas myristoleic acid, PC(14:1(9Z)/P-18:1(9Z)), and glucosylceramide tended to decrease in hairy roots, suggesting remodeling of membrane lipid-related metabolites in transformed roots. Among terpenoids, 3-epioleanolic acid, linalool, manool, sweroside, and procurcumenol showed clear accumulation trends in RUBY hairy roots (Figure 4C). Flavonoids generally showed decreased accumulation, with cynaroside, isoquercitrin, kaempferol-3-O-galactoside, astragalin, and hyperoside all reduced in both K599 and RUBY hairy roots (Figure 4D). Phenylpropanoids displayed divergent response patterns. For example, (E)-ferulic acid increased in K599 but decreased in RUBY, whereas 4-methoxycinnamyl alcohol and N-feruloyloctopamine were up-regulated in both types of hairy roots (Figure 4E). Among amino acids, 1-aminocyclopropane-1-carboxylic acid, L-arginine, L-citrulline, L-ornithine, L-proline, L-glutamine, and N-acetylornithine were generally up-regulated in both K599 and RUBY hairy roots (Figure 4F), indicating marked changes in amino acid and nitrogen-related metabolism during hairy root formation.

Overall, changes in these five representative metabolite classes indicated that both K599 and RUBY hairy roots formed metabolic states distinct from WT roots. The most prominent changes involved amino acid/nitrogen metabolism, lipid-related metabolism, terpenoid-related metabolism, and phenylpropanoid and flavonoid metabolism. In addition, several metabolites showed further changes in RUBY vs. K599, indicating RUBY-associated metabolic variation in the hairy root background.

### 2.5. Genome-Wide Identification and Characterization of HpRHL-like bHLH Genes

To identify RHL-related candidate genes potentially involved in root hair development in *H. pedunculosum*, the RHL1 protein sequences from *Arabidopsis thaliana* and *L. japonicus* were used as references for homology searches against the whole-genome protein dataset of *H. pedunculosum*. After homology screening and conserved domain verification, a total of 80 HpRHL-like bHLH candidate genes were identified. These genes were named HpRHL01–HpRHL80 according to their physical positions on chromosomes (Table S4). Chromosomal localization analysis showed that the 80 HpRHL-like genes were distributed across all 10 chromosomes of *H. pedunculosum*, but their distribution was uneven (Figure S1). Chr02 contained the largest number of HpRHL-like genes, with 13 members, whereas Chr10 contained the fewest, with only four members. Further analysis identified four obvious tandemly duplicated gene clusters located at the lower end of Chr01, the upper region of Chr02, the lower half of Chr04, and the top region of Chr09. The genes within these clusters were closely and continuously arranged, suggesting that tandem duplication may have contributed to the local expansion of the HpRHL-like gene family.

**Figure 5:**
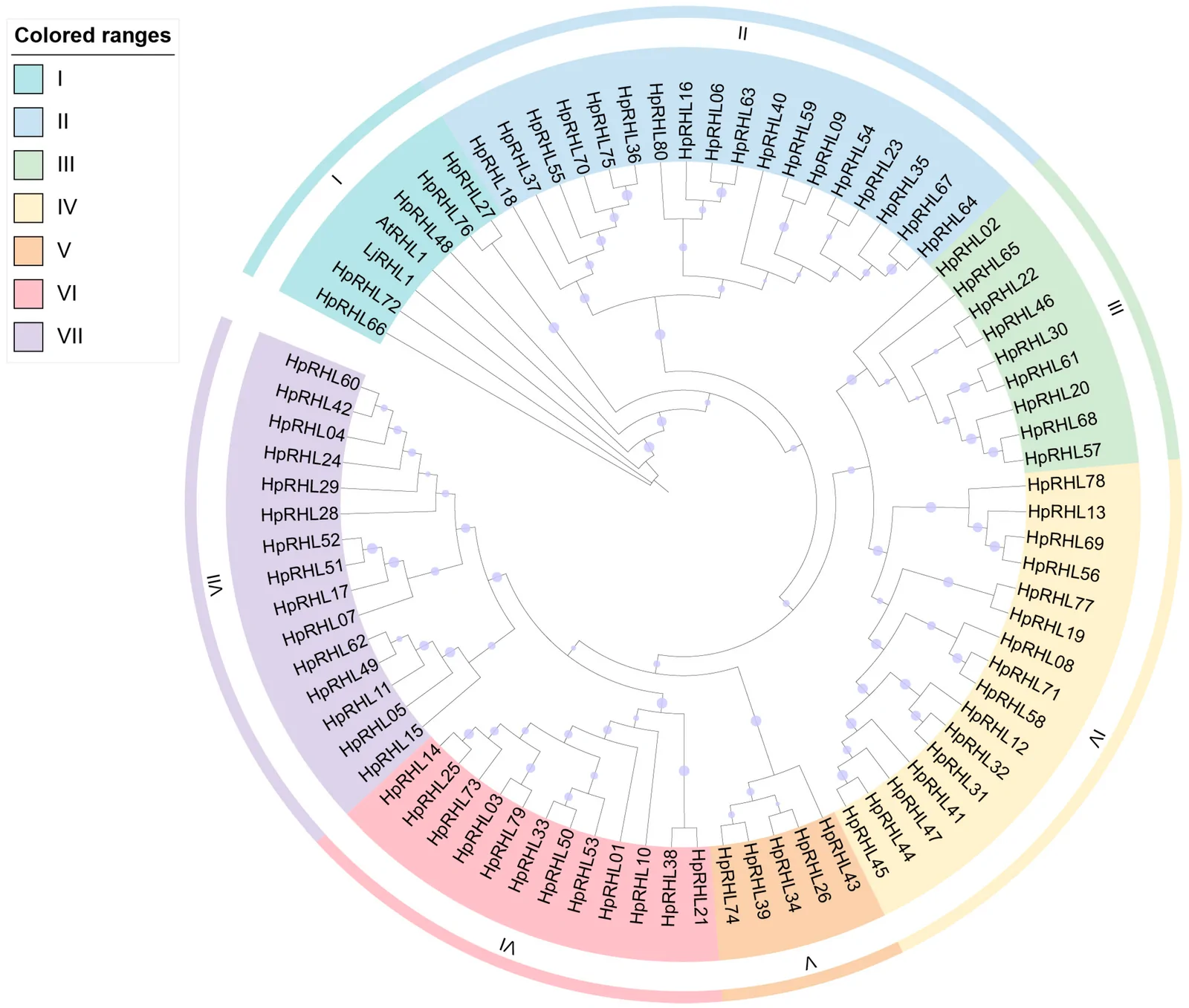
Phylogenetic analysis of HpRHL-like bHLH proteins in *H. pedunculosum*. The phylogenetic tree was constructed using 80 HpRHL-like proteins from *H. pedunculosum*, AtRHL1 from *Arabidopsis thaliana*, and LjRHL1 from *Lotus japonicus*. HpRHL-like proteins were tentatively divided into seven phylogenetic groups, designated Group I–Group VII, according to their clustering patterns. Different groups are indicated by different colors. This grouping was based primarily on phylogenetic relationships and does not represent a strict functional classification based on known subfamilies in model plants. AtRHL1 and LjRHL1 clustered within Group I.

To clarify the evolutionary relationships of HpRHL-like genes, the 80 HpRHL-like proteins were aligned together with AtRHL1 from *A. thaliana* and LjRHL1 from *L. japonicus*, and a phylogenetic tree was constructed (Figure 5). Based on the clustering pattern of the phylogenetic tree, the 80 HpRHL-like bHLH proteins were tentatively divided into seven phylogenetic groups, designated Group I–Group VII. This grouping was used for subsequent comparative analyses of gene structure, conserved motifs, and chromosomal distribution. Among these groups, five HpRHL-like proteins, namely HpRHL27, HpRHL48, HpRHL66, HpRHL72, and HpRHL76, clustered close to AtRHL1 and LjRHL1, suggesting that these members may be evolutionarily related to RHL1-like proteins from model plants. At the same time, several HpRHL-like members formed species-specific branches, indicating lineage-specific diversification within *H. pedunculosum*.

Synteny analysis further provided insight into the potential expansion mechanisms of the HpRHL-like gene family. Intraspecific synteny analysis revealed multiple syntenic HpRHL-like gene pairs within the *H. pedunculosum* genome, with a particularly clear syntenic relationship between Chr02 and Chr09 (Figure S2), suggesting that segmental duplication may have contributed to the expansion of some HpRHL-like genes. Interspecific synteny analysis showed several conserved syntenic blocks between *H. pedunculosum* and *A. thaliana* or *L. japonicus*. Some HpRHL-like genes were syntenic with genomic regions containing AtRHL1- or LjRHL1-related genes (Figure S3), suggesting that these genes may retain certain evolutionary relationships across different plant species.

Gene structure, conserved motif, and conserved domain analyses were further performed for the HpRHL-like genes. The results showed that the exon–intron structures of HpRHL-like genes were diverse, although most members contained only a small number of introns. Conserved motif analysis identified 10 major motifs, designated Motif 1–Motif 10. Motif 1 and Motif 2 were present in most HpRHL-like proteins, suggesting that they may represent conserved core structural features of this protein group. By contrast, Motif 3–Motif 10 showed differential distributions among different phylogenetic groups, indicating structural divergence within the family (Figure S4). Conserved domain annotation showed that HpRHL-like proteins mainly contained bHLH-related domains, and different members contained distinct domain types or domain features, such as bHLH_AtBPE_like and bHLH_MYC_N. These results suggest that HpRHL-like members may have undergone functional diversification.

### 2.6. Subcellular Localization and Expression Patterns of Representative HpRHL-like Genes

**Figure 6:**
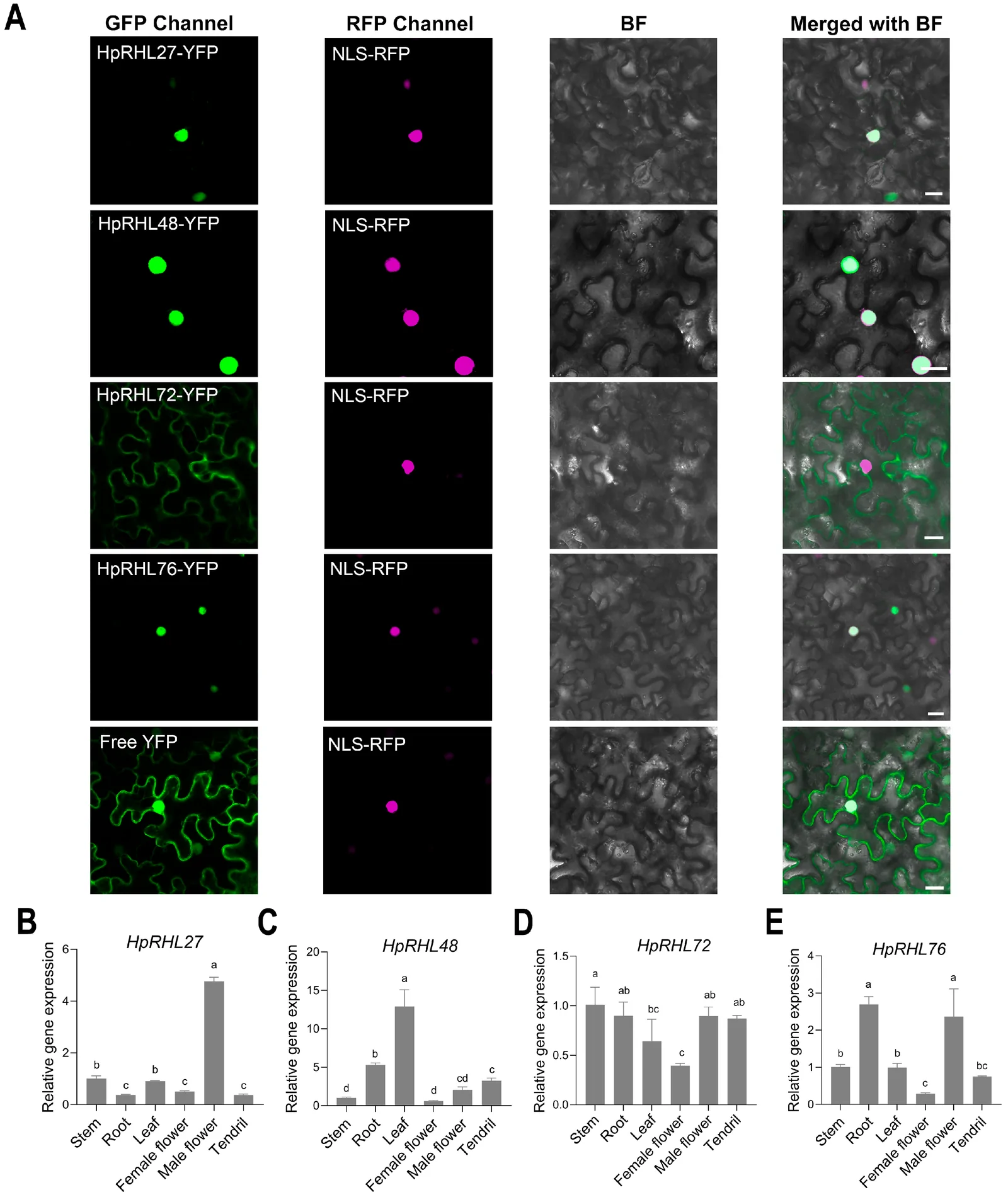
Subcellular localization and tissue expression patterns of four representative HpRHL-like genes. (**A**) Subcellular localization of four representative HpRHL-like proteins in *Nicotiana benthamiana* leaves. YFP fluorescence detected in the GFP channel, RFP fluorescence, bright-field (BF), and merged images are shown. The RFP signal of the nuclear marker is displayed in magenta as a pseudo-color to improve contrast and readability. Scale bars = 20 μm. (**B**–**E**) RT-qPCR analysis of *HpRHL27* (**B**), *HpRHL48* (**C**), *HpRHL72* (**D**), and *HpRHL76* (**E**) in different tissues of *H. pedunculosum*. Data are shown as the mean ± SE. Different lowercase letters indicate significant differences at *p* < 0.05 (*n* = 3).

To further investigate the potential functions of HpRHL-like genes, we focused on candidates that were phylogenetically close to the functionally characterized LjRHL1. Among these candidates, four representative genes, *HpRHL27*, *HpRHL48*, *HpRHL72*, and *HpRHL76*, were successfully cloned and selected for subcellular localization and tissue expression analyses. The four genes were individually fused to YFP under the control of the CaMV 35S promoter, generating p35S::HpRHL27-YFP, p35S::HpRHL48-YFP, p35S::HpRHL72-YFP, and p35S::HpRHL76-YFP constructs, and transiently expressed in *Nicotiana benthamiana* leaves. HpRHL27-YFP, HpRHL48-YFP, and HpRHL76-YFP predominantly overlapped with the nuclear marker, whereas HpRHL72-YFP showed a distinct peripheral fluorescence pattern (Figure 6). Further co-localization analysis with the plasma membrane marker OsRac3-mCherry showed partial signal overlap, supporting partial plasma membrane-associated localization of HpRHL72 (Figure S5).

Tissue expression patterns of the four genes were analyzed by RT-qPCR in stems, roots, leaves, female flowers, male flowers, and tendrils of *H. pedunculosum* (Figure 6B–E). *HpRHL27* was most highly expressed in male flowers, with moderate expression in stems and leaves. *HpRHL48* showed its highest expression in leaves and was also detectable in roots. *HpRHL72* exhibited relatively high expression in roots, stems, male flowers, and tendrils, whereas its expression was lowest in female flowers. *HpRHL76* showed relatively high expression in roots and male flowers, with root expression significantly higher than that in stems, leaves, female flowers, and tendrils. Considering both its phylogenetic proximity to functionally characterized RHL proteins and its relatively high expression in roots, *HpRHL76* was selected as a representative candidate for subsequent proof-of-concept functional analysis.

### 2.7. Application of the RUBY-Assisted Hairy Root System for Preliminary Functional Analysis of HpRHL76

**Figure 7:**
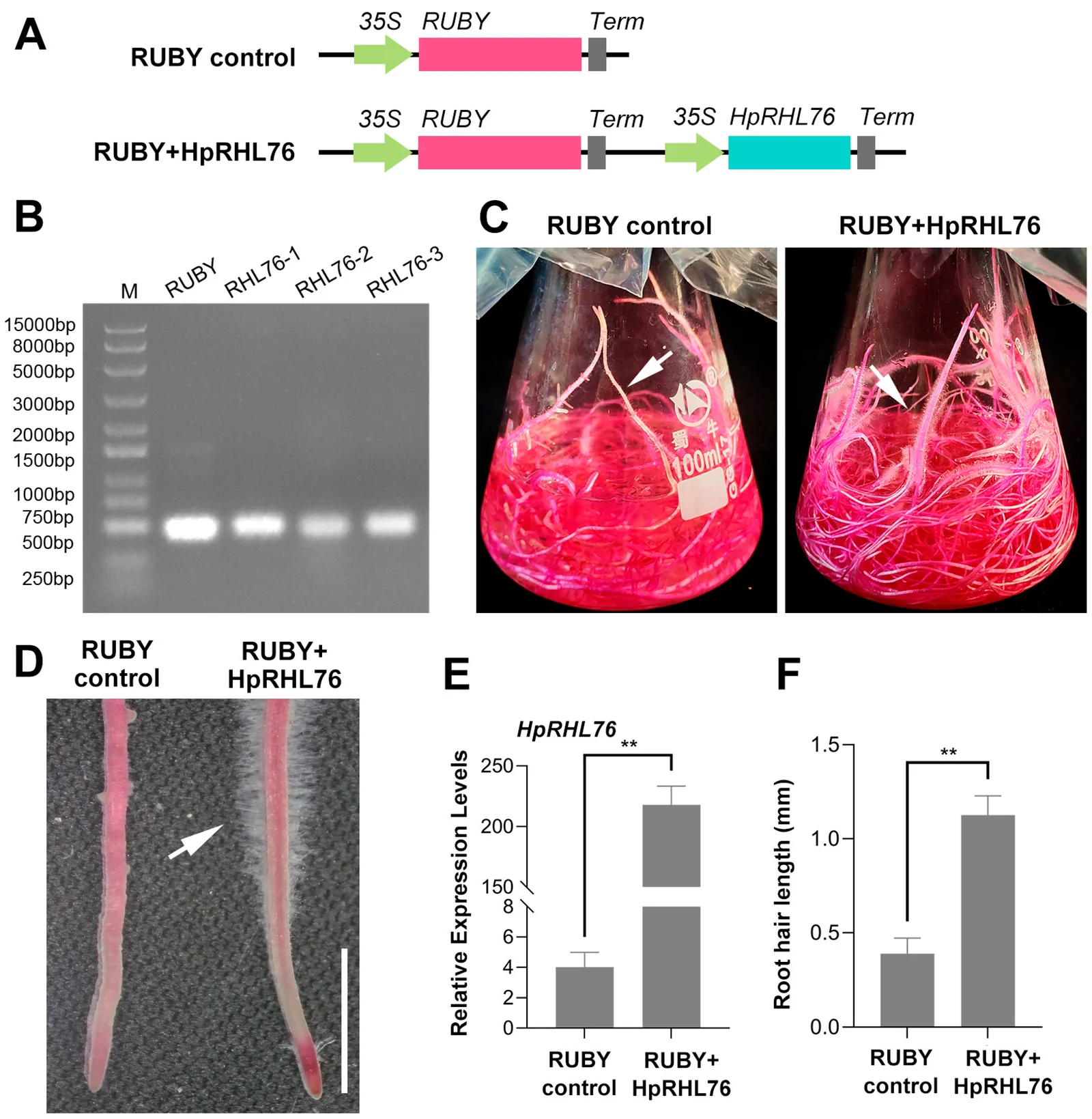
Preliminary functional analysis of *HpRHL76* using the RUBY-assisted hairy root system. (**A**) Schematic representation of the RUBY control construct and the *HpRHL76* dual-expression construct. The RUBY control construct contains the 35S::RUBY expression cassette, whereas the *HpRHL76* construct contains two independent expression cassettes, 35S::RUBY and 35S::HpRHL76. (**B**) PCR detection of *rolB* in candidate hairy roots. RUBY indicates hairy roots transformed with the RUBY control construct; RHL76-1, RHL76-2, and RHL76-3 indicate three independent hairy root lines transformed with the *HpRHL76* construct. (**C**,**D**) Representative root hair phenotypes of hairy roots transformed with the *HpRHL76* construct and the RUBY control construct. White arrows indicate root hair regions. Scale bars = 1 cm. (**E**) RT-qPCR analysis of relative *HpRHL76* transcript levels in RUBY control and *HpRHL76*-overexpressing hairy roots. (**F**) Quantitative comparison of root hair length between RUBY control and *HpRHL76*-overexpressing hairy roots. Data are presented as mean ± SE. ** indicate significant differences at *p* < 0.01 (Student’s *t*-test) (*n* = 3).

*HpRHL76* was subsequently used as a proof-of-concept candidate based on the combined phylogenetic and tissue-expression evidence described above. An *HpRHL76* overexpression construct carrying the RUBY visual reporter was generated (Figure 7A), while a construct containing only the 35S::RUBY expression cassette served as the control. The two constructs were separately introduced into *R. rhizogenes* K599 and used to transform aseptic seedlings of *H. pedunculosum*. The visible red pigmentation conferred by RUBY allowed rapid preliminary identification of candidate positive hairy roots. Three independent hairy root lines carrying the *HpRHL76* construct were subsequently selected for molecular verification and phenotypic analysis.

PCR amplification of *rolB* produced the expected fragment in both RUBY control hairy roots and hairy roots carrying the *HpRHL76* construct, confirming their *R. rhizogenes*-induced origin (Figure 7B). RT-qPCR analysis further showed that the relative transcript abundance of *HpRHL76* was markedly higher in hairy roots carrying the *HpRHL76* overexpression construct than in the RUBY control (Figure 7E), confirming successful overexpression of *HpRHL76* in the transformed hairy roots.

Phenotypic analysis showed longer root hairs in *HpRHL76*-overexpressing hairy roots than in the RUBY control (Figure 7C,D). Quantitative analysis showed that the mean root hair length increased from approximately 0.4 mm in the RUBY control to approximately 1.1 mm in *HpRHL76*-overexpressing hairy roots, with a significant difference between the two groups (Figure 7F). These results provide preliminary evidence that increased *HpRHL76* expression is associated with enhanced root hair elongation in *H. pedunculosum* and support the applicability of the RUBY-assisted hairy root system for preliminary assessment of root development-related candidate genes.

## 3. Discussion

### 3.1. A Rapid Aseptic Seedling Establishment System Provides a Stable Explant Source for Genetic Transformation in H. pedunculosum

The seeds of *H. pedunculosum* have a hard seed coat, a well-developed cuticular layer, and strong dormancy, which often result in low germination rates, asynchronous germination, and a prolonged seedling production period during conventional soaking, germination, and cultivation processes. These features represent one of the major constraints limiting experimental research and artificial propagation of this species [32]. In addition, the rough and uneven seed surface of *H. pedunculosum* may retain microorganisms during aseptic germination, increasing the risk of contamination. Therefore, establishing a stable and controllable aseptic seedling production system that allows convenient contamination monitoring is a prerequisite for subsequent hairy root induction and genetic transformation experiments.

In this study, three germination systems, namely the solid medium method (Sm), vermiculite medium method (Vm), and cotton-supported liquid culture method (Clm), were compared for aseptic seedling establishment (Figure 1). The conventional Sm method not only showed the lowest germination rate but also usually required removal of the seed coat to promote germination, which increased pretreatment time and contamination risk and made it unsuitable for the rapid production of large numbers of aseptic explants. In contrast, both Vm and Clm significantly improved seed germination efficiency, indicating that optimization of the germination substrate can effectively alleviate the difficulty of aseptic seedling production in *H. pedunculosum*. Although Vm showed the highest numerical germination rate and the shortest germination period, its opaque granular substrate was not conducive to the early detection of latent contamination. Once microorganisms spread within the gaps between vermiculite particles, the risk of cross-contamination during subsequent culture may increase, thereby affecting the stable supply of aseptic explants.

The Clm system uses moist sterile cotton as a supporting substrate, providing a stable water environment for seed germination while allowing convenient observation of seed status during culture. The white cotton background also facilitates visual recognition of fungal or bacterial contamination, enabling contaminated material to be removed before subsequent experiments. Although Vm showed the numerically highest germination rate, its germination rate was not significantly different from that of Clm. In our experimental workflow, Clm therefore offered a practical advantage because contamination could be monitored more readily than in vermiculite-based culture. However, contamination rate and seedling survival were not systematically quantified in the present study, and the two methods therefore cannot be compared statistically with respect to these parameters. Accordingly, Clm should be regarded as a practical choice for aseptic seedling establishment in this study, based on its comparable germination performance, ease of contamination monitoring, and compatibility with subsequent hairy root induction, rather than as a universally optimal culture method.

### 3.2. The RUBY-Assisted Hairy Root System Provides a Practical Platform for Genetic Transformation in H. pedunculosum

As an endangered medicinal plant, *H. pedunculosum* has long lacked an efficient genetic transformation system, limiting functional genomic and metabolic engineering studies [33]. In this study, we established a RUBY-assisted hairy root transformation system based on *R. rhizogenes*-mediated induction. Comparison of wounded stem-segment immersion (SSI) and microsyringe-mediated stem-base inoculation (MSI) showed that MSI was more suitable for aseptic seedlings of *H. pedunculosum* (Figure 2). Further optimization identified an injection volume of 3 μL, a bacterial density of OD600 = 0.9, and 300 mg/L cefotaxime sodium as appropriate conditions, resulting in a maximum transformation efficiency of 93.33%, defined as the proportion of inoculated seedlings producing at least one RUBY-positive hairy root. These results indicate that targeted stem-base inoculation provides an efficient and reproducible approach for hairy root induction in this species.

The incorporation of RUBY represents an important practical advantage of the system. Conventional reporters such as GFP and GUS generally require fluorescence imaging or destructive histochemical staining [34,35], whereas RUBY generates visible red betalain pigmentation that allows rapid and non-destructive identification of candidate transformed roots [20,36]. In our system, RUBY-positive hairy roots could therefore be distinguished directly by eye, substantially simplifying preliminary screening before downstream molecular and phenotypic analyses. Because RUBY-associated betalain biosynthesis may itself influence the metabolic background, however, its use in metabolic studies should be accompanied by appropriate transformation controls, as further evaluated in this study.

Hairy root systems have been widely applied in medicinal plants for either metabolic engineering or rapid gene functional analysis. For example, pathway engineering in *Salvia miltiorrhiza* increased tanshinone accumulation, whereas an optimized *Astragalus membranaceus* system achieved an induction rate of 80.1% and supported enhanced astragaloside production [16,37]. Within Cucurbitaceae, K599-mediated hairy root systems have been established in cucumber and melon and subsequently used for CRISPR/Cas9-based analysis of RHL-related genes [27,38]. These studies demonstrate the broad utility of hairy roots, but direct numerical comparison of transformation efficiencies should be made cautiously because different studies use different experimental units and calculation criteria. For example, the cucumber study reported the proportion of explants producing hairy roots, whereas the 93.33% efficiency reported here represents the proportion of inoculated seedlings producing at least one RUBY-positive hairy root. Thus, the contribution of the present system lies not simply in its transformation efficiency, but in the integration of species-adapted aseptic seedling establishment, RUBY-based visual screening, evaluation of transformation-associated metabolic variation, and subsequent proof-of-concept candidate gene assessment.

The workflow developed here may also provide a useful framework for other difficult-to-transform herbaceous plants and cucurbit species. Previous studies in cucumber, melon, and watermelon indicate that transformation performance can vary with genotype, explant type and developmental stage, *R. rhizogenes* strain, bacterial density, inoculation method, co-cultivation conditions, and antibiotic treatment [27,38,39]. RUBY performance and its potential metabolic effects may likewise require species-specific evaluation. Therefore, the principal transferable feature of this study is the integrated experimental strategy rather than the specific transformation parameters.

### 3.3. Hairy Root Formation Is Associated with Broad Metabolic Changes in H. pedunculosum

Hairy root systems are widely used for secondary metabolite production, metabolic engineering, and gene functional studies in medicinal plants because of their rapid growth, relative genetic stability, and independence from whole-plant regeneration [10,17]. However, hairy roots are not metabolically equivalent to wild-type roots. Ri T-DNA integration, *rol* gene activity, and in vitro culture conditions can influence plant development, hormone homeostasis, and metabolism [40]. Recent studies in Cucurbitaceae have similarly shown that *R. rhizogenes*-mediated hairy root formation can be accompanied by substantial transcriptomic and metabolomic changes [31]. Such transformation-associated effects should therefore be considered when hairy roots are used for metabolic studies.

The metabolomic profiles obtained in this study were consistent with this view (Figure 3 and Figure 4). The K599 vs. WT comparison revealed extensive metabolic differences associated with the hairy root state and *R. rhizogenes*-mediated transformation. In addition to the independent pairwise comparisons, K599 and RUBY samples were pooled as the HRs group only as a complementary analysis to characterize the overall metabolic background of hairy roots relative to WT, rather than to identify changes specifically shared by the two hairy root types. The HRs vs. WT comparison showed pronounced alterations in amino acids, sugars, organic acids, and lipids. Amino acid-related metabolites were predominantly increased, whereas several monosaccharides decreased and a number of disaccharides or oligosaccharides accumulated, suggesting substantial changes in carbon and nitrogen metabolism. Several lipid-related metabolites were also reduced, indicating alterations in lipid composition associated with the hairy root state.

KEGG enrichment analysis further indicated changes in amino acid biosynthesis and degradation, galactose metabolism, starch and sucrose metabolism, flavone and flavonol biosynthesis, monoterpenoid biosynthesis, plant hormone biosynthesis, and ABC transporters (Figure 3H). These changes may reflect altered carbon and nitrogen allocation, cellular metabolic demands, and specialized metabolic activity in rapidly growing transformed roots, although their precise biological significance remains to be experimentally established. In particular, changes in primary metabolism could potentially influence precursor availability and metabolic flux toward medicinally relevant specialized metabolites.

However, the widely targeted metabolomic analysis used here did not clearly annotate the characteristic cucurbitacins or lignans of *H. pedunculosum*. The present data therefore cannot determine whether hairy root formation causes sustained increases or decreases in the accumulation of these key medicinal compounds. Future studies combining targeted LC–MS/MS quantification using authentic standards, time-course metabolomics, matched transcriptomic analyses, and functional validation of candidate biosynthetic genes will be required to clarify the long-term metabolic consequences of hairy root formation and to evaluate its suitability for metabolic engineering of medicinally important compounds.

### 3.4. RUBY-Associated Transformation Introduces Additional Metabolic Variation in Hairy Roots

Despite its practical advantages for visual screening, RUBY-expressing hairy roots remained metabolically distinguishable from vector-free K599-induced hairy roots. A total of 169 differential metabolites were identified in the RUBY vs. K599 comparison, involving lipid-related metabolites, terpenoid-related metabolites, phenylpropanoids, flavonoids, and other metabolic classes. These results indicate that introduction of the RUBY construct is associated with additional metabolic variation beyond the general metabolic background of *R. rhizogenes*-induced hairy roots.

This observation is particularly relevant when RUBY is applied in metabolomics or metabolic engineering, because the reporter-associated transformation should not automatically be assumed to be metabolically neutral. Importantly, however, the differences between RUBY and K599 hairy roots cannot be attributed exclusively to RUBY expression. Binary-construct introduction, T-DNA integration, betalain biosynthesis, transformation-associated variation, and differences among independently generated hairy root lines may all contribute to the observed metabolic profiles. The present results should therefore be interpreted as RUBY-associated metabolic differences rather than direct metabolic effects of RUBY expression.

These findings also highlight the importance of including appropriate transformation-background controls in RUBY-based metabolic studies. The inclusion of vector-free K599-induced hairy roots in the present study allowed the general metabolic background associated with hairy root formation to be distinguished from additional variation associated with introduction of the RUBY construct and/or RUBY expression. Thus, although RUBY provides a convenient visual screening strategy, its potential influence on the metabolic background should be considered when it is applied to specialized metabolite studies or metabolic engineering.

### 3.5. Preliminary Functional Assessment of HpRHL76 Supports the Applicability of the RUBY-Assisted Hairy Root System

To evaluate the applicability of the RUBY-assisted hairy root system for preliminary gene functional analysis in *H. pedunculosum*, we selected RHL-like bHLH transcription factors as proof-of-concept candidates because of their well-established association with root hair development and the readily observable phenotypes resulting from altered activity. RHL/RSL-related bHLH factors participate in root hair initiation, elongation, and epidermal cell differentiation in several plant species [30,41]. For example, disruption of *LjRHL1* in *Lotus japonicus* causes defective root hair development, and related bHLH factors have also been implicated in root hair regulation in *Arabidopsis* and other plants [30,42]. Similar applications have recently been reported in other cucurbits, where CRISPR/Cas9-mediated disruption of *CsbHLH66*/*CsbHLH82* in cucumber and *CmRHL1* in melon resulted in pronounced root hair defects in transformed hairy roots [27,38]. In contrast, the present study used *HpRHL76* overexpression and observed enhanced root hair elongation, providing complementary but preliminary evidence for the utility of hairy root systems in rapid assessment of root development-related genes in cucurbits.

We identified 80 HpRHL-like bHLH candidates from the *H. pedunculosum* genome and tentatively classified them into seven phylogenetic groups (Figure 5). This grouping was based primarily on phylogenetic relationships and was intended to facilitate comparative analysis rather than to imply strict functional classification. Among the representative candidates examined further, *HpRHL27*, *HpRHL48*, *HpRHL72*, and *HpRHL76* showed distinct tissue expression patterns (Figure 6). *HpRHL76* was selected for subsequent proof-of-concept analysis based on the combined evidence of its phylogenetic proximity to functionally characterized RHL proteins and its relatively high expression in roots. HpRHL76-YFP predominantly overlapped with the nuclear marker in *N. benthamiana*, consistent with its predicted role as a transcription factor.

Using the RUBY-assisted hairy root system, candidate roots carrying the *HpRHL76* overexpression construct could be rapidly identified for subsequent analysis (Figure 7). RT-qPCR confirmed markedly increased *HpRHL76* transcript abundance in the overexpression roots, and quantitative phenotypic analysis showed that mean root hair length increased from approximately 0.4 mm in RUBY control hairy roots to approximately 1.1 mm in *HpRHL76*-overexpressing hairy roots. These results provide preliminary evidence that increased *HpRHL76* expression is associated with enhanced root hair elongation in *H. pedunculosum*, consistent with the established involvement of RHL/RSL-related bHLH factors in root hair development [30,42]. Importantly, however, overexpression alone is insufficient to establish the precise biological function of *HpRHL76*. Future loss-of-function approaches, such as gene knockout or silencing, together with complementation analysis, will be required to determine whether *HpRHL76* is necessary for root hair elongation and to clarify its regulatory role.

Overall, the *HpRHL76* experiment serves primarily as a proof of concept for the developed transformation system rather than as definitive functional characterization of this gene. The results demonstrate the feasibility of combining RUBY-based visual screening with molecular and quantitative phenotypic analyses for preliminary assessment of candidate genes directly in *H. pedunculosum* hairy roots. For a species lacking an efficient whole-plant transformation system, this platform provides a practical basis for future studies of root development, candidate gene function, and specialized metabolism.

### 3.6. Limitations

Several limitations of this study should be acknowledged. First, the metabolic differences observed among WT, vector-free K599-induced hairy roots, and RUBY-expressing hairy roots cannot be attributed to a single causal factor. Hairy root induction, Ri T-DNA/*rol* gene effects, binary-construct introduction, and RUBY-associated betalain biosynthesis may all contribute to the observed variation. Therefore, the changes in amino acid/nitrogen metabolism, sugar metabolism, lipid-related metabolites, flavonoids, and terpenoid-related pathways should be interpreted as transformation- and reporter-associated metabolic differences rather than direct effects of RUBY expression. Their biological significance, particularly for medicinally relevant specialized metabolites, will require further confirmation by targeted metabolite quantification and additional molecular evidence.

Second, the functional assessment of *HpRHL76* was based primarily on overexpression, and further loss-of-function or complementary genetic evidence will be required to establish its precise biological role. Root hair density was not quantified because extensive overlap prevented reliable counting under the imaging conditions used. In addition, hairy roots may not fully reproduce the developmental and metabolic physiology of intact roots or whole plants. Finally, because matched transcriptomic and metabolomic datasets were not generated from the same experimental samples, potential gene–metabolite regulatory relationships could not be reliably resolved. Future studies integrating matched multi-omics datasets with targeted genetic and metabolic validation will be needed to address these limitations.

## 4. Materials and Methods

### 4.1. Seed Source and Aseptic Seedling Establishment of H. pedunculosum

The *H. pedunculosum* seeds used in this study were obtained from a cultivated medicinal plant population maintained at the cultivation base of Tibet Rhodiola Pharmaceutical Holding Company in Nyingchi, Xizang, China. The material was not derived from a formally defined accession, inbred line, or genetically uniform germplasm line. A voucher specimen (No. H-200215-1), identified by Professor Qi Zhao, was deposited at Chengdu University. All experimental materials were derived from the same cultivated germplasm source.

In this study, three methods were used to establish aseptic seedlings of *H. pedunculosum*: the solid medium culture method (Sm), cotton-supported liquid culture method (Clm), and vermiculite medium method (Vm). The seed surface sterilization procedure was performed as follows. First, the seed surface was thoroughly scrubbed with neutral detergent and rinsed with tap water 5–6 times until no visible foam remained. The washed seeds were first treated with warm water at 50 °C for 30 min, held at room temperature for 6 h, and then immersed in 200 mg/L penicillin solution for 12 h. Surface disinfection was conducted under aseptic conditions in a laminar flow cabinet. The seeds were sterilized with 75% ethanol for 1.5 min, rinsed once with sterile water, treated with 5% sodium hypochlorite for 5 min, and finally rinsed five to six times with sterile water to eliminate residual sterilizing agents.

The solid medium culture method was performed with appropriate modifications based on a previously reported aseptic culture method for *H. pedunculosum* [43]. For the cotton-supported liquid culture method, sterile cotton was used as the supporting substrate, whereas sterilized vermiculite was used as the cultivation substrate for the vermiculite medium method. Both methods used 1/2 MS liquid medium (Coolaber, Beijing, China) [44] for moistening or irrigation, and the seed sterilization and sowing procedures were kept consistent. In the Clm system, sterilized seeds were placed on sterile cotton saturated with 1/2 MS liquid medium and incubated in darkness at 20–25 °C for 7–10 d. Germinated seedlings were then transferred to a 16 h light/8 h dark photoperiod for further growth. In the Vm system, sterilized seeds were planted in autoclaved vermiculite, and substrate moisture was maintained by adding 1/2 MS liquid medium. Other culture conditions were identical to those used for Clm.

Throughout the culture period, seed germination timing, contamination occurrence, and seedling growth status were monitored and documented. Germination rate was determined by dividing the number of germinated seeds by the total number of seeds inoculated. Differences in germination rate among the three culture methods were analyzed using one-way ANOVA followed by Tukey’s multiple comparison test in GraphPad Prism 8.0.1, with *p* < 0.05 considered statistically significant. This value was then used to evaluate and compare the germination performance and operational suitability of the three aseptic seedling establishment methods.

### 4.2. Vector Construction

The pCAMBIA1300-35S::RUBY vector used for RUBY-assisted hairy root induction was generated by modifying the UBQ::RUBY plasmid. The UBQ::RUBY plasmid was obtained from Addgene (www.addgene.org). The RUBY coding region was PCR-amplified and cloned into the pCAMBIA1300-35S backbone by homologous recombination, resulting in the CaMV 35S promoter-driven pCAMBIA1300-35S::RUBY construct (Figure S6). This construct was subsequently introduced into *R. rhizogenes* strain K599 (Weidibio, Shanghai, China) for inducing RUBY-positive hairy roots in *H. pedunculosum*.

Vectors for subcellular localization analysis were constructed as follows. The coding sequences of *HpRHL27*, *HpRHL48*, *HpRHL72*, and *HpRHL76* were amplified without their stop codons. The amplified fragments were then individually inserted into the pCAMBIA1300-35S-YFP vector to generate C-terminal YFP fusion constructs, namely pCAMBIA1300-35S::HpRHL27-YFP, pCAMBIA1300-35S::HpRHL48-YFP, pCAMBIA1300-35S::HpRHL72-YFP, and pCAMBIA1300-35S::HpRHL76-YFP. Each subcellular localization vector was transferred into *A. tumefaciens* strain GV3101-p19 and subsequently used for transient infiltration of *N. benthamiana* leaves.

The *HpRHL76* overexpression construct used for functional validation was generated by modifying the pCAMBIA1300-35S::RUBY vector. Through two rounds of homologous recombination, the 35S::HpRHL76 expression cassette and the corresponding terminator were introduced into the vector while retaining the 35S::RUBY expression cassette. The resulting dual-expression vector, pCAMBIA1300-35S::RUBY-35S::HpRHL76 (Figure S7), was introduced into *R. rhizogenes* strain K599 for the induction of RUBY-labeled *H. pedunculosum* hairy roots carrying the *HpRHL76* overexpression construct. All recombinant vectors were verified by PCR amplification and Sanger sequencing before transformation into the corresponding bacterial strains.

### 4.3. Hairy Root Induction and Transformation

A frozen stock of *R. rhizogenes* strain K599 was revived in YEP liquid medium and incubated overnight at 28 °C on a shaker at 200 rpm. The overnight culture was then harvested by centrifugation at 5000 rpm for 3 min at room temperature. After removing the supernatant, the bacterial pellet was resuspended in liquid MS medium and adjusted to different OD600 values according to the experimental requirements for infection of *H. pedunculosum* explants.

Two methods were used for hairy root induction in *H. pedunculosum*: microsyringe-mediated stem-base inoculation (MSI) and wounded stem-segment immersion (SSI). For MSI, a defined volume of K599 bacterial suspension was drawn using a microsyringe (Shanghai Bolige Industry and Trade Co., Ltd., Shanghai, China) and injected into the stem base of aseptic *H. pedunculosum* seedlings. In the SSI method, aseptic seedlings were sectioned into 2–5 mm stem pieces and precultured on 1/2 MS solid medium for 2 d. These stem pieces were then infected by immersion in the K599 suspension for 15 min, after which they were transferred to 1/2 MS medium and co-cultivated in darkness for 2–3 d.

Following co-cultivation, the infected explants were moved to 1/2 MS medium containing Cefotaxime Na Salt (LABLEAD, Beijing, China) to inhibit overgrowth of K599. The Cef stock solution was sterilized by filtration through a 0.22 μm membrane and added to the autoclaved medium after the medium temperature had decreased sufficiently. To determine suitable conditions for hairy root induction in *H. pedunculosum*, a series of single-factor assays was conducted to examine the influence of inoculation method, injection volume, Cef concentration, and bacterial density on transformation efficiency. The inoculation methods included MSI and SSI; the injection volumes were set at 3, 6, and 9 μL; the Cef concentrations were set at 200, 300, 400, and 500 mg/L; and the bacterial densities were set at OD600 = 0.3, 0.5, 0.7, 0.9, and 1.1. Each treatment consisted of 10 experimental units, and three independent experimental replicates were performed, resulting in 30 experimental units per treatment. Hairy root transformation efficiency was calculated as the percentage of inoculated experimental units that produced at least one RUBY-positive hairy root; multiple RUBY-positive roots arising from the same experimental unit were counted as a single successful transformation event.

Under the optimized conditions, *H. pedunculosum* explants were infected with K599 carrying either the 35S::RUBY construct or the dual-expression construct containing independent 35S::RUBY and 35S::HpRHL76 expression cassettes. Red hairy roots emerging from the explant base were regarded as putative RUBY-positive roots and were excised when they reached approximately 5 cm in length. These roots were then transferred to liquid 1/2 MS medium and maintained on a shaker for further culture. Putative positive roots were screened visually based on RUBY-derived red pigmentation, after which genomic DNA was extracted and *rolB* was amplified by PCR to confirm their origin from *R. rhizogenes*-mediated hairy root induction [45]. For the K599 group used in metabolomic analysis, hairy roots were induced under the same optimized conditions using *R. rhizogenes* K599 without an introduced binary vector and were confirmed by PCR detection of *rolB*. The primers used for molecular identification are listed in Table S1.

For root hair phenotypic analysis, RUBY control hairy roots and *HpRHL76*-overexpressing hairy roots were observed and photographed using a stereomicroscope (Tieniu, Shangrao, China). Root hairs in comparable regions near the root tip were measured from calibrated images using ImageJ software (bundled with 64-bit Java 8), and mean root hair length was calculated for statistical comparison between the two groups.

### 4.4. Widely Targeted Metabolomic Analysis of Hairy Roots

Fresh samples of wild-type roots (WT), *R. rhizogenes* K599-induced hairy roots without an introduced binary vector (K599), and RUBY-expressing hairy roots induced by K599 carrying the 35S::RUBY construct (RUBY) of *H. pedunculosum* were collected, immediately frozen in liquid nitrogen, and sent to Gene Denovo Biotechnology Co. (Guangzhou, China) for widely targeted metabolomic analysis. Three independent biological replicates were included for each group. Each biological replicate consisted of pooled root tissues collected from multiple seedlings subjected to the same treatment, with equal amounts of fresh tissue contributed by each seedling to reduce the influence of individual variation. The pooled material from each biological replicate was then divided into aliquots for LC–MS/MS analysis.

Freeze-dried root samples were ground into powder, and 50 mg of each sample was extracted with 1 mL of methanol:acetonitrile:water (2:2:1, *v*/*v*/*v*). After extraction, sonication, and centrifugation, the supernatant was collected, dried, and reconstituted in acetonitrile:water (1:1, *v*/*v*) for LC–MS/MS analysis. Equal aliquots from all samples were mixed to generate quality control (QC) samples, which were used to monitor the stability and reproducibility of the metabolomic analysis.

Widely targeted metabolomic profiling was performed by Gene Denov Biotechnology Co. (Guangzhou, China) using a UPLC–ESI–MS/MS platform. Chromatographic separation was carried out on a Waters Acquity I-Class PLUS system equipped with a Waters HSS-T3 column, and metabolites were detected using a QTRAP 6500+ mass spectrometer (SCIEX, Framingham, MA, USA) in multiple reaction monitoring (MRM) mode under both positive and negative ionization modes. Metabolite identification and quantification were performed based on the GENE DENOVO in-house database, using retention time, precursor and product ions, and optimized mass spectrometric parameters. The relative abundance of each metabolite was calculated from the corresponding chromatographic peak area. *p*-values were corrected for multiple testing, and false discovery rate (FDR) values were calculated.

For visualization and hierarchical clustering, metabolite abundance data were standardized by Z-score transformation and plotted using the R package pheatmap (v1.0.12). Differential metabolites among different sample groups were identified using variable importance in projection (VIP) values from the OPLS-DA model together with statistical significance based on Student’s *t*-test. Metabolites with VIP ≥ 1 and *p* ≤ 0.05 were considered differentially accumulated. The identified differential metabolites were further subjected to KEGG pathway annotation and enrichment analysis [46].

### 4.5. Identification and Phylogenetic Analysis of HpRHL-like bHLH Genes

For genome-wide identification of HpRHL-like genes, chromosome-level genomic resources of *H. pedunculosum*, including the genome sequence, gene annotation, CDS sequences, and predicted protein sequences, were obtained from our previously published genome dataset [47]. The AtRHL1 and LjRHL1 protein sequences were used as queries to search the predicted proteome of *H. pedunculosum* by BLASTP using TBtools II (v2.525) [48]. Candidate proteins were initially screened based on sequence similarity and subsequently examined for conserved domains. Only proteins containing conserved bHLH-related domains were retained and designated as HpRHL-like bHLH proteins. A total of 80 candidates were identified and named *HpRHL01*–*HpRHL80* according to their chromosomal positions.

The full-length amino acid sequences of the 80 HpRHL-like proteins, together with AtRHL1 and LjRHL1, were used for phylogenetic analysis. A maximum-likelihood (ML) phylogenetic tree was constructed using IQ-TREE (v2.3.6), with the best-fit amino acid substitution model automatically selected by ModelFinder. Branch support was evaluated using 1000 bootstrap replicates. The resulting tree was visualized and annotated using the Interactive Tree Of Life (iTOL) platform. Based on the clustering pattern of the ML tree, the 80 HpRHL-like bHLH proteins were tentatively classified into seven phylogenetic groups, designated Groups I–VII.

### 4.6. Chromosomal Distribution and Synteny Analysis of HpRHL-like Genes

The chromosomal locations of HpRHL-like genes were extracted from the *H. pedunculosum* genome annotation file and plotted using TBtools II [48]. Tandem duplication events were inferred based on HpRHL-like genes that were adjacent or closely clustered on the same chromosome.

To investigate gene duplication and conservation, synteny analyses were performed using the MCScanX-related modules in TBtools II. Syntenic relationships within the *H. pedunculosum* genome were visualized with a Circos plot. For cross-species comparisons, genome FASTA files and GFF3 annotation files from *H. pedunculosum*, *A. thaliana*, and *L. japonicus* were used to identify conserved syntenic blocks. The resulting interspecific syntenic relationships were displayed using the Dual Synteny Plot module in TBtools II.

### 4.7. Analysis of Gene Structures, Conserved Motifs, and Protein Domains of HpRHL-like Genes

Conserved protein domains were annotated using NCBI Batch CD-Search. Motif prediction was conducted in TBtools II using a MEME-based workflow, and up to 10 conserved motifs were searched for each HpRHL-like protein. The MEME output file, conserved domain results, phylogenetic tree file, HpRHL-like gene ID list, and *H. pedunculosum* genome annotation file were then imported into the Gene Structure View (Advanced) module in TBtools II to integrate and visualize the phylogenetic relationships, exon–intron structures, conserved motifs, and conserved domain distributions of HpRHL-like genes.

### 4.8. RT-qPCR Analysis of Gene Expression

For tissue-specific expression analysis, stem, root, leaf, male flower, female flower, and tendril samples of *H. pedunculosum* were collected and immediately frozen in liquid nitrogen. Total RNA was isolated using a plant RNA extraction kit (Foregene Biotechnology, Chengdu, China). Genomic DNA removal and first-strand cDNA synthesis were performed using HiFiScript gDNA Removal RT MasterMix (Cwbio, Taizhou, Jiangsu, China) following the manufacturer’s protocol.

RT-qPCR was conducted on a Bio-Rad CFX96 real-time PCR system (Bio-Rad, Shanghai, China) using a SYBR-based qPCR premix (Cwbio, Taizhou, Jiangsu, China). Gene-specific primers were designed with Primer Premier 6, and their sequences are provided in Table S1. Each reaction was performed in a 10 μL volume containing SYBR premix, gene-specific primers, cDNA template, and nuclease-free water. The amplification was carried out for 40 cycles according to the recommended program of the reagent manufacturer.

The previously reported *HpActin* gene was used as the internal control [49]. Relative transcript levels were calculated using the 2^−ΔΔCT^ method. Three biological replicates were included for each tissue. Data are shown as the mean ± SE. Statistical analysis was performed using GraphPad Prism 8.0.1, and differences among tissues were evaluated by one-way ANOVA followed by Tukey’s multiple comparison test. Differences were considered significant at *p* < 0.05.

For verification of *HpRHL76* overexpression in hairy roots, three independent biological replicates were analyzed for both the RUBY control and *HpRHL76*-overexpression groups, with each biological replicate derived from an independently transformed seedling. Total RNA was extracted separately from each replicate, followed by genomic DNA removal and cDNA synthesis. Relative *HpRHL76* transcript abundance was calculated using *HpActin* as the internal reference and the RUBY control as the calibrator. Differences between the two groups were evaluated using Student’s *t*-test.

### 4.9. Subcellular Localization Analysis

A rapid and reliable transient co-expression system has not yet been established for *H. pedunculosum* hairy roots, making this system less suitable for preliminary subcellular localization assays requiring simultaneous expression of fluorescent fusion proteins and organelle markers. Therefore, *N*. *benthamiana* leaves, which provide a well-established and highly reproducible transient expression system, were used for initial localization analysis of HpRHL-like proteins.

For subcellular localization assays, the YFP fusion constructs pCAMBIA1300-35S::HpRHL27-YFP, pCAMBIA1300-35S::HpRHL48-YFP, pCAMBIA1300-35S::HpRHL72-YFP, and pCAMBIA1300-35S::HpRHL76-YFP were separately transformed into *A. tumefaciens* GV3101-p19. Positive colonies were cultured overnight in YEP liquid medium containing the appropriate antibiotics at 28 °C and 200 rpm. The bacterial cells were then harvested, resuspended in MMA infiltration buffer, and adjusted to OD600 = 0.5.

For transient expression, each *Agrobacterium* suspension carrying an HpRHL-YFP construct was mixed at a 1:1 ratio with the suspension carrying the nuclear marker RFP-NLS [50]. To further assess the peripheral fluorescence pattern observed for HpRHL72, HpRHL72-YFP was additionally co-expressed with the plasma membrane marker OsRac3-mCherry [51]. After standing in the dark at room temperature for 2–3 h, the mixtures were infiltrated into the abaxial side of leaves of 4–6-week-old *N. benthamiana* plants using a needleless syringe. The pCAMBIA1300-35S::YFP vector was used as the control. Infiltrated plants were grown under normal conditions, and leaf tissues from the infiltrated areas were sampled 48 h later for fluorescence imaging.

Fluorescence signals were detected using a Nikon AX R confocal laser scanning microscope (Nikon, Tokyo, Japan). YFP fluorescence was detected using the GFP channel, whereas RFP-NLS and OsRac3-mCherry signals were detected using the corresponding red fluorescence channel. Co-localization patterns between HpRHL-YFP fusion proteins and the nuclear or plasma membrane markers were evaluated from the merged fluorescence images. At least three independent infiltration regions were observed for each subcellular localization experiment, and representative fluorescence images were selected for presentation.

## 5. Conclusions

In this study, we established a RUBY-assisted hairy root transformation platform for *H. pedunculosum* and evaluated the metabolic background associated with hairy root induction and RUBY-associated transformation. As a proof of concept, *HpRHL76* overexpression was associated with significantly increased root hair length, providing preliminary evidence for its potential involvement in root hair elongation and supporting the applicability of the platform for initial candidate gene assessment. This integrated system provides a practical basis for future functional genomic studies, investigation and metabolic engineering of medicinally relevant specialized metabolites, and may also serve as a methodological reference for genetic studies in other difficult-to-transform medicinal plant species.

## Data Availability

The original contributions presented in this study are included in the article/Supplementary Materials. Further inquiries can be directed to the corresponding authors.

## Supplementary Materials

The following supporting information can be downloaded at https://www.mdpi.com/article/10.3390/plants15162514/s1, Figure S1. Chromosomal distribution of HpRHL-like genes in *H. pedunculosum*; Figure S2. Intraspecific synteny analysis of HpRHL-like gene family members in *H. pedunculosum*; Figure S3. Interspecific synteny analysis of RHL-like gene family members among *H. pedunculosum*, *Arabidopsis thaliana*, and *Lotus japonicus*; Figure S4. Conserved motif, conserved domain, and gene structure analyses of HpRHL-like genes in *H. pedunculosum*; Figure S5. Subcellular localization of HpRHL72 and its partial co-localization with a plasma membrane marker in *Nicotiana benthamiana* leaves; Figure S6. Schematic map of the pCAMBIA1300-35S::RUBY vector used for RUBY-assisted hairy root transformation; Figure S7. Schematic map of the dual-expression pCAMBIA1300-35S::RUBY-35S::*HpRHL76* vector used for *HpRHL76* overexpression in hairy roots; Table S1. Sequences of primers used in this study; Table S2. Differential metabolites of sugars, organic acids, amino acids, and lipids identified in HRs vs. WT; Table S3. Selected differential metabolites of terpenoids, phenylpropanoids, amino acids, flavonoids, and lipids used for focused metabolite analysis; Table S4. List of HpRHL-like genes and reference RHL1 proteins used for phylogenetic analysis.

## Abbreviations

The following abbreviations are used in this manuscript:

ANOVA	Analysis of variance
BF	Bright field
bHLH	Basic helix-loop-helix
Cef	Cefotaxime sodium
Clm	Cotton-supported liquid culture method
GFP	Green fluorescent protein
HRs	Pooled hairy root samples
MSI	Microsyringe-mediated stem-base inoculation
NCBI	National Center for Biotechnology Information
NLS	Nuclear localization signal
PCA	Principal component analysis
QC	Quality control
RFP	Red fluorescent protein
RHL	ROOT HAIRLESS
RUBY	Betalain biosynthesis-based visual reporter; RUBY-expressing hairy roots in sample labels
SSI	Stem-segment immersion
VIP	Variable importance in projection
WT	Wild-type
YFP	Yellow fluorescent protein
