# Supplementary material for: A RUBY-Assisted Hairy Root Transformation Platform for Metabolomic Profiling and Preliminary Functional Analysis of HpRHL76 in Herpetospermum pedunculosum
Source: Plants (Basel). 2026 Aug 20;15(16):2514. doi: 10.3390/plants15162514 (PMC13516302; doi:10.3390/plants15162514)
Supplement: Supplementary file 1 [file plants-15-02514-s001.zip › Supplementary Figures.pdf]

Supplementary Figures

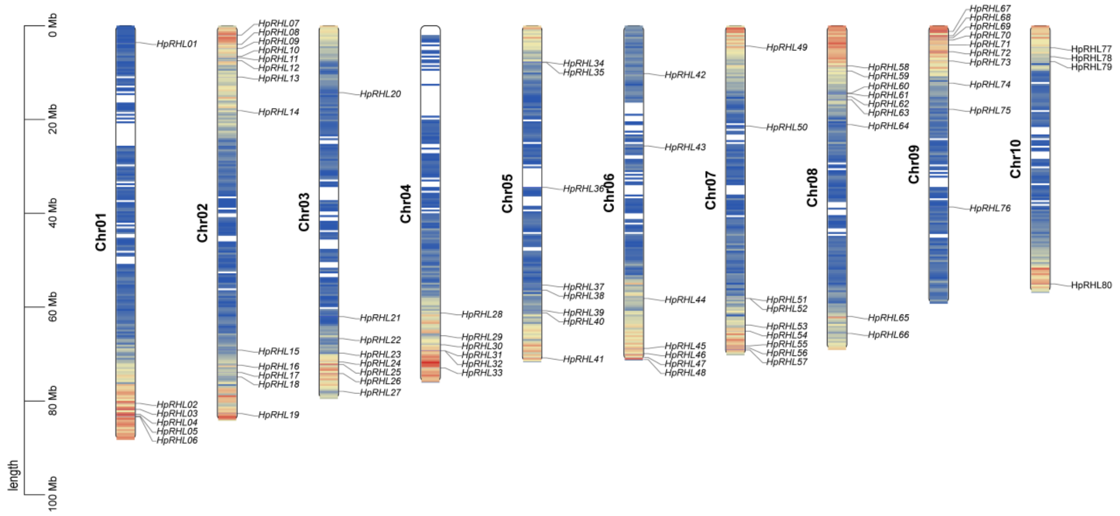

Figure S1. Chromosomal distribution of HpRHL-like genes in *H. pedunculosum*.

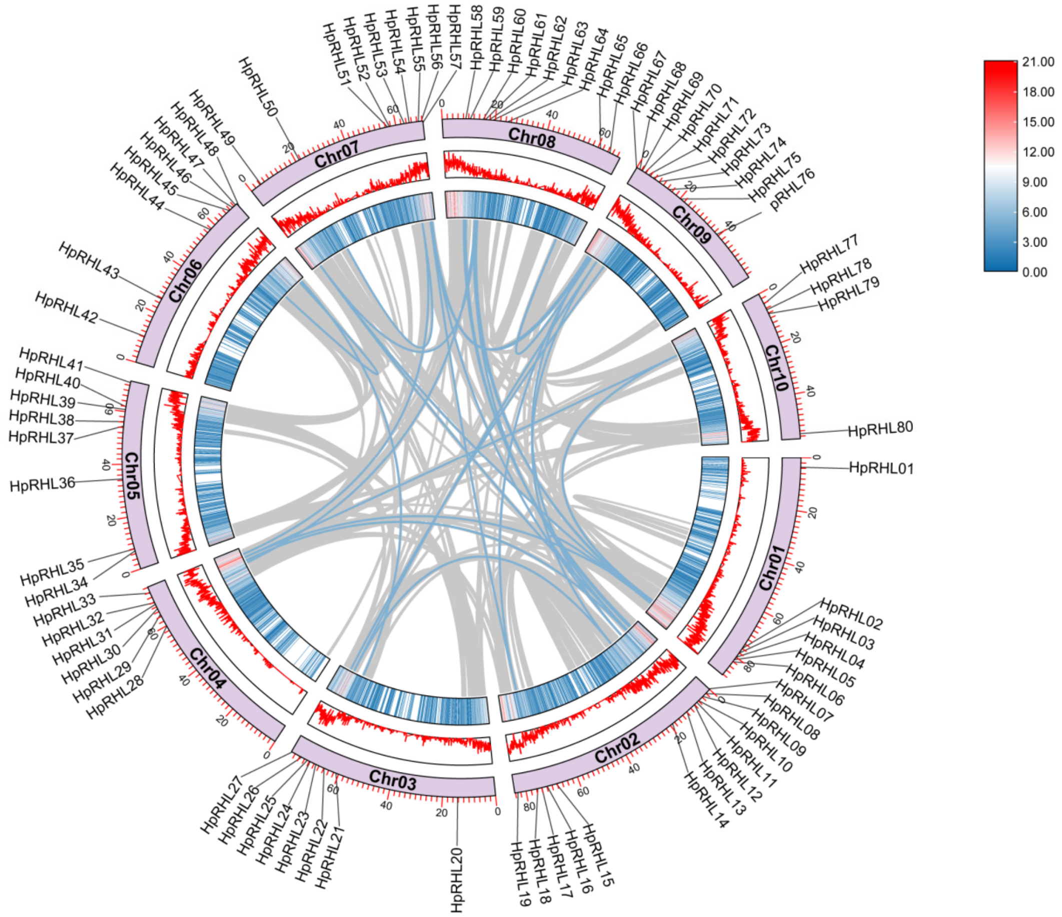

Figure S2. Intraspecific synteny analysis of HpRHL-like gene family members in *H. pedunculosum*.

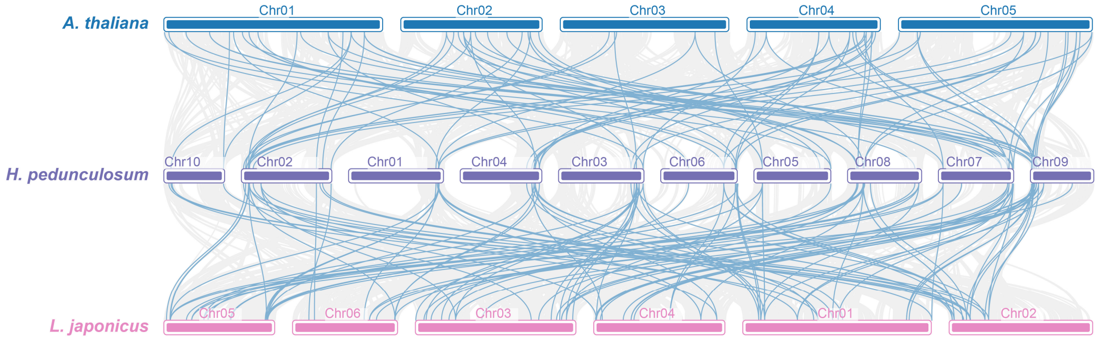

Figure S3. Interspecific synteny analysis of RHL-like gene family members among *H. pedunculosum*, *Arabidopsis thaliana*, and *Lotus japonicus*.

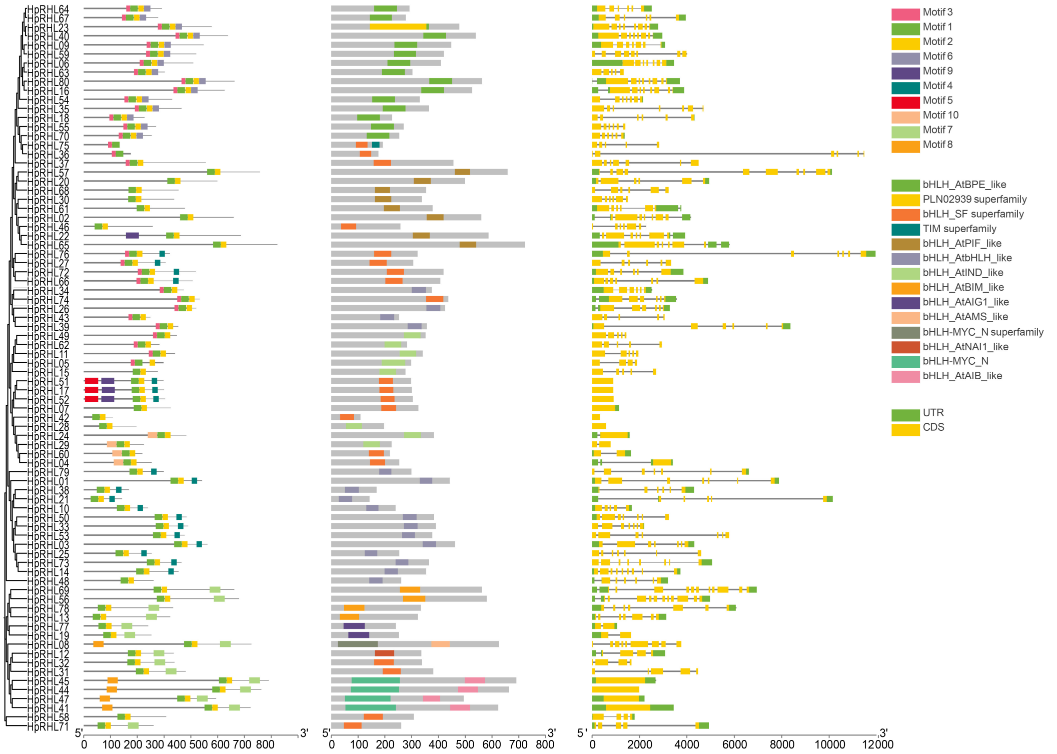

Figure S4. Conserved motif, conserved domain, and gene structure analyses of HpRHL-like genes in *H. pedunculosum*.

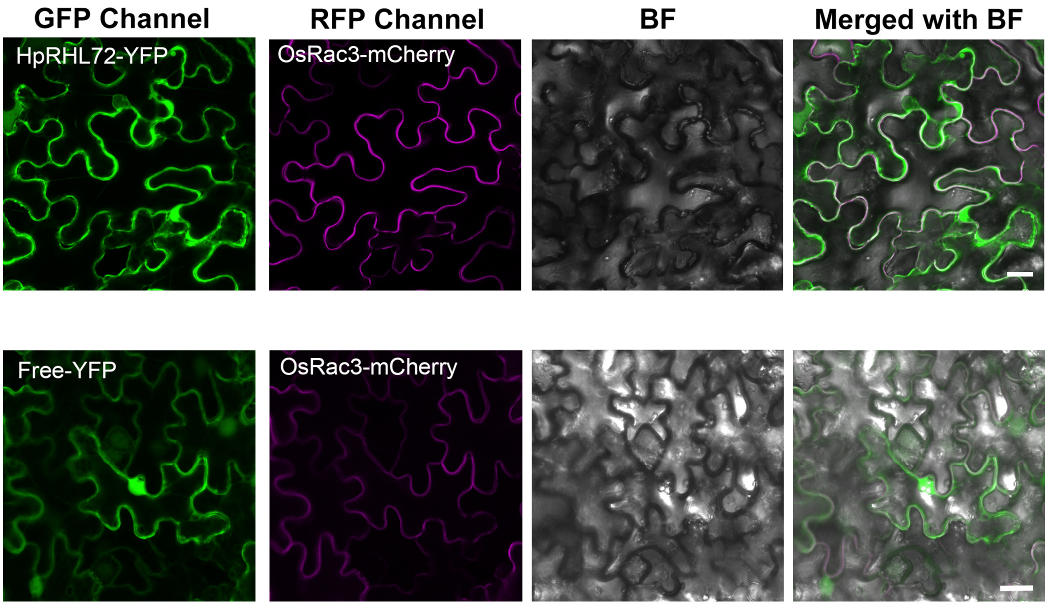

Figure S5. Subcellular localization of HpRHL72 and its partial co-localization with a plasma membrane marker in *Nicotiana benthamiana* leaves.

YFP fluorescence detected in the GFP channel, RFP fluorescence from the plasma membrane marker, bright-field (BF), and merged images are shown. The RFP signal is displayed in magenta as a pseudo-color to improve contrast and readability. Partial overlap between the HpRHL72-YFP signal and the plasma membrane marker (OsRac3-mCherry) is visible in the merged images. Scale bars = 20 μm.

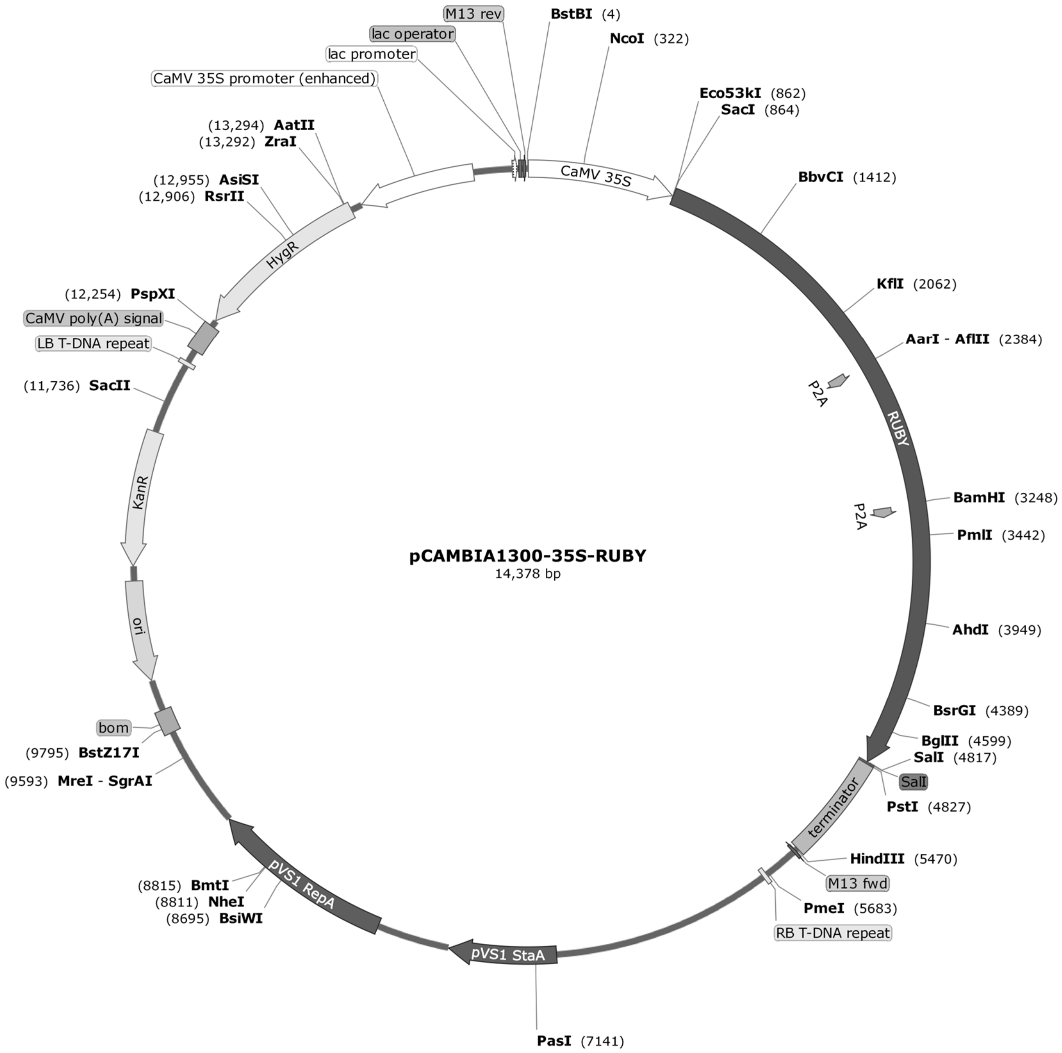

Figure S6. Schematic map of the pCAMBIA1300-35S::RUBY vector used for RUBY-assisted hairy root transformation.

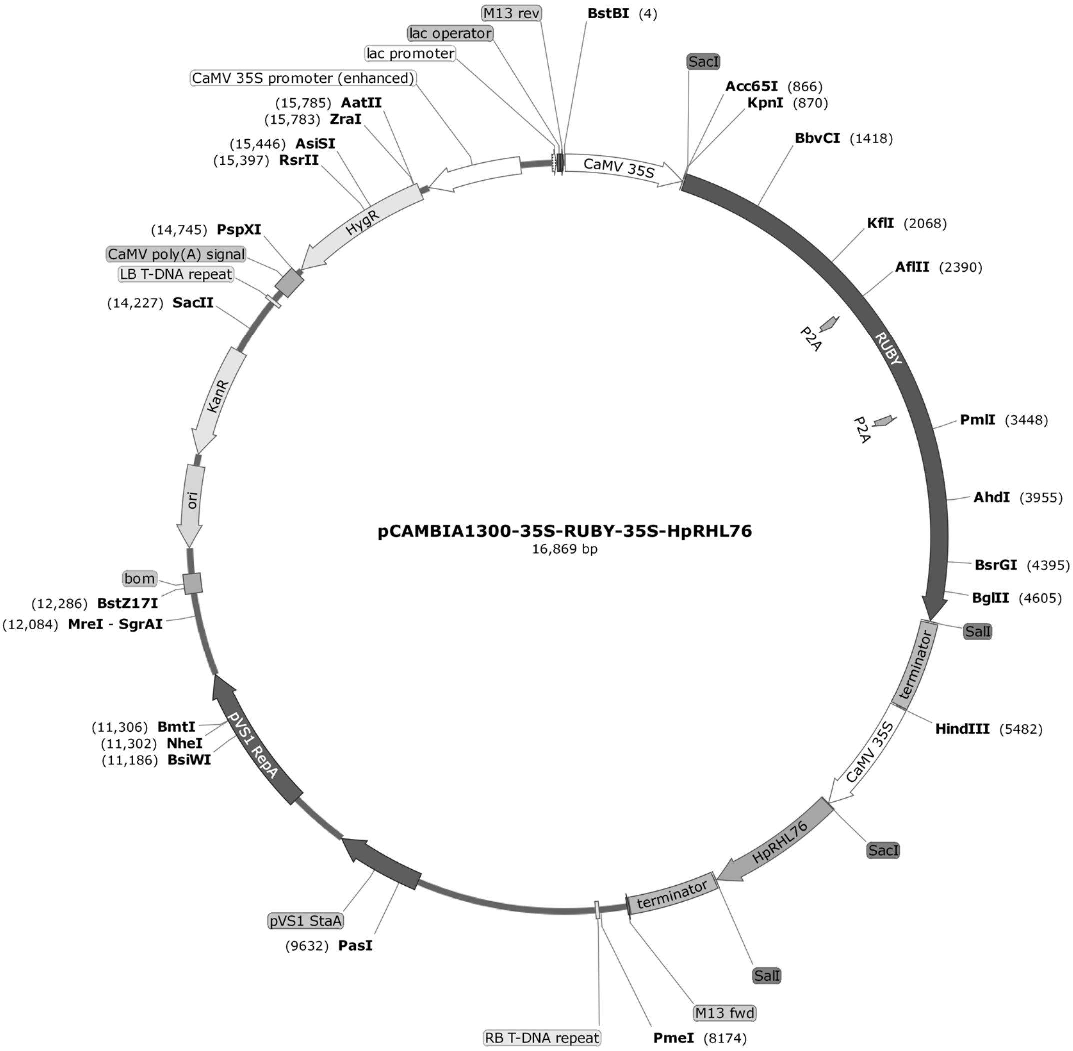

Figure S7. Schematic map of the dual-expression pCAMBIA1300-35S::RUBY-35S::*HpRHL76* vector used for *HpRHL76* overexpression in hairy roots.
